# Supplementary material for: Developing Ni single-atom sites in carbon nitride for efficient photocatalytic H2O2 production
Source: Nat Commun. 2023 Nov 6;14:7115. doi: 10.1038/s41467-023-42887-y (PMC10628073; doi:10.1038/s41467-023-42887-y)
Supplement: Supplementary file 1 — Supplementary Information [file 41467_2023_42887_MOESM1_ESM.pdf]

Supplementary Information

Developing Ni single-atom sites in carbon nitride for efficient photocatalytic H_2O_2 production

Xu Zhang[1,#], Hui Su[2,3,#], Peixin Cui[4], Yongyong Cao[5], Zhenyuan Teng[6], Qitao Zhang[7], Yang Wang[8], Yibo Feng[1], Ran Feng[1], Jixiang Hou[1], Xiyuan Zhou[1], Peijie Ma[1], Hanwen Hu[1], Kaiwen Wang[1], Cong Wang[1], Liyong Gan[8], Yunxuan Zhao[9], Qinghua Liu*[2], Tierui Zhang*[9], Kun Zheng*[1]

[1]Beijing Key Laboratory of Microstructure and Properties of Solids, Faculty of Materials and Manufacturing, Beijing University of Technology, Beijing, 100124, China. [2]National Synchrotron Radiation Laboratory, University of Science and Technology of China, Hefei 230029, Anhui, China. [3]College of Chemistry and Chemical Engineering, Hunan Normal University, Changsha 410081, Hunan, China. [4]Key Laboratory of Soil Environment and Pollution Remediation, Institute of Soil Science, Chinese Academy of Sciences, 210008, Nanjing, China. [5]College of Biological, Chemical Science and Engineering, Jiaxing University, Jiaxing, Zhejiang, 314001, China. [6]School of Chemistry, Chemical Engineering and Biotechnology, Nanyang Technological University, Singapore 637459, Singapore. [7]International Collaborative Laboratory of 2D Materials for Optoelectronics Science and Technology of Ministry of Education, Institute of Microscale Optoelectronics, Shenzhen University, Shenzhen 518060, China. [8]College of Physics and Institute of Advanced Interdisciplinary Studies, Chongqing University, Chongqing, 400044, China. [9]Key Laboratory of Photochemical Conversion and Optoelectronic Materials, Technical Institute of Physics and Chemistry, Chinese Academy of Sciences, Beijing, China. [#] These authors contributed equally: Xu Zhang, Hui Su.

✉e-mail: qhliu@ustc.edu.cn; tierui@mail.ipc.ac.cn; kunzheng@bjut.edu.cn.

This file includes:

Supplementary Figures 1-39.

Supplementary Tables 1-7.

Supplementary References.

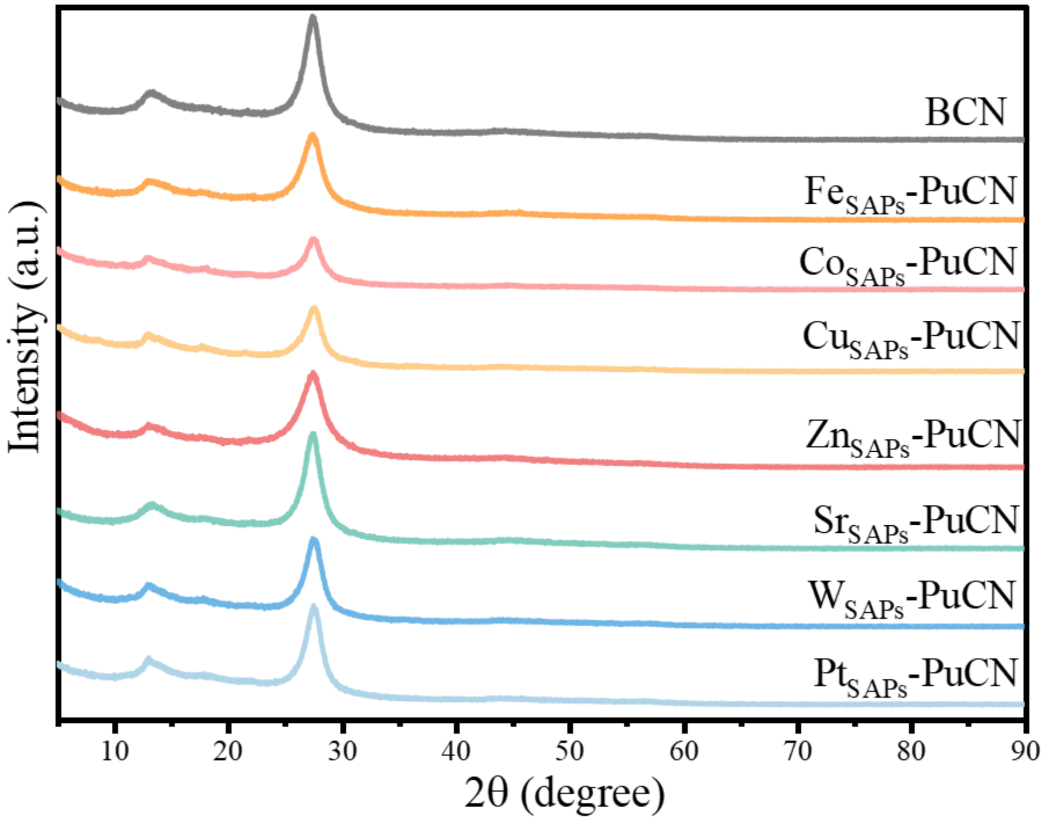

Supplementary Figure 1. XRD patterns of BCN and M_{SAPs}-PuCN (M =Fe, Co, Cu, Zn, Sr, W, Pt).

In **Supplementary Figure 1**, the original bulk g-C_3N_4 (BCN) displays two XRD diffraction peaks at 2θ = 13.4° and 27.4°, assigned to the (100) and (002) planes, which represent the in-plane packing of heptazine units and interlayer stacking of g-C_3N_4 sheets, respectively. M_{SAPs}-PuCN still guarantee the structural characteristic peaks of g-C_3N_4 and no corresponding metal diffraction peaks appear. This proves from the side that there are no metal particles or clusters in these samples.

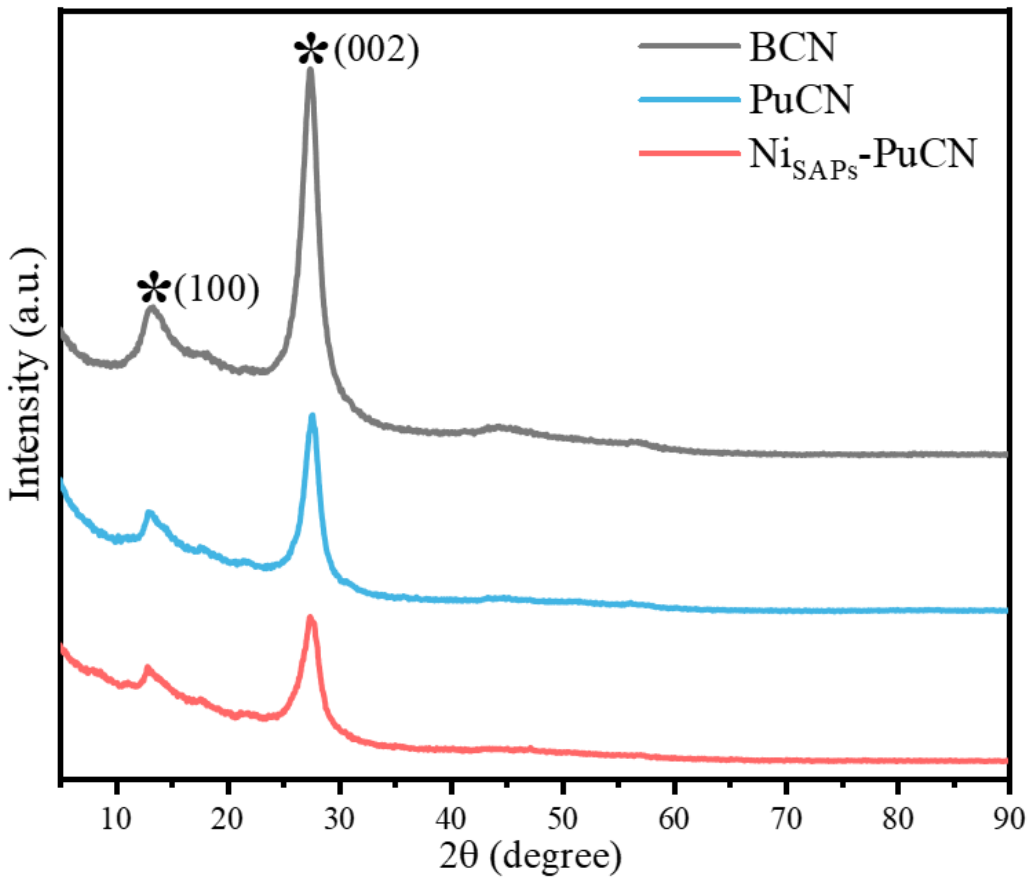

Supplementary Figure 2. XRD patterns of BCN, PuCN, and Ni_{SAPs}-PuCN.

In **Supplementary Figure 2,** Compared with BCN, it can be clearly seen that the (002) peaks of PuCN and Ni_{SAPs}-PuCN become weaker, which indicates that the stacking strength between g-C_3N_4 layers decreases, corresponding to its porous ultrathin structure.

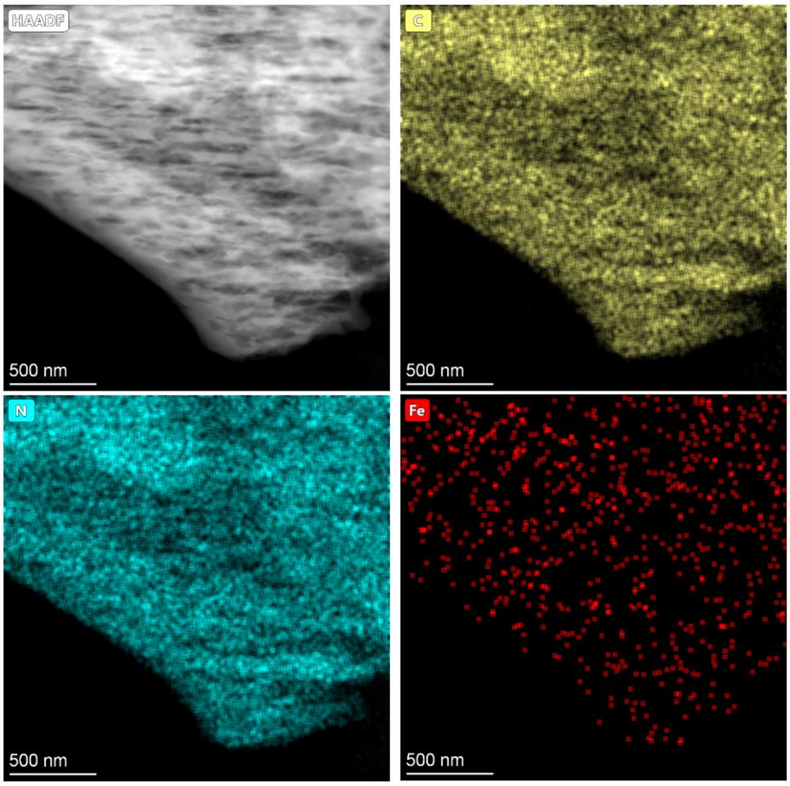

Supplementary Figure 3. EDS mapping of Fe_{SAPs}-PuCN.

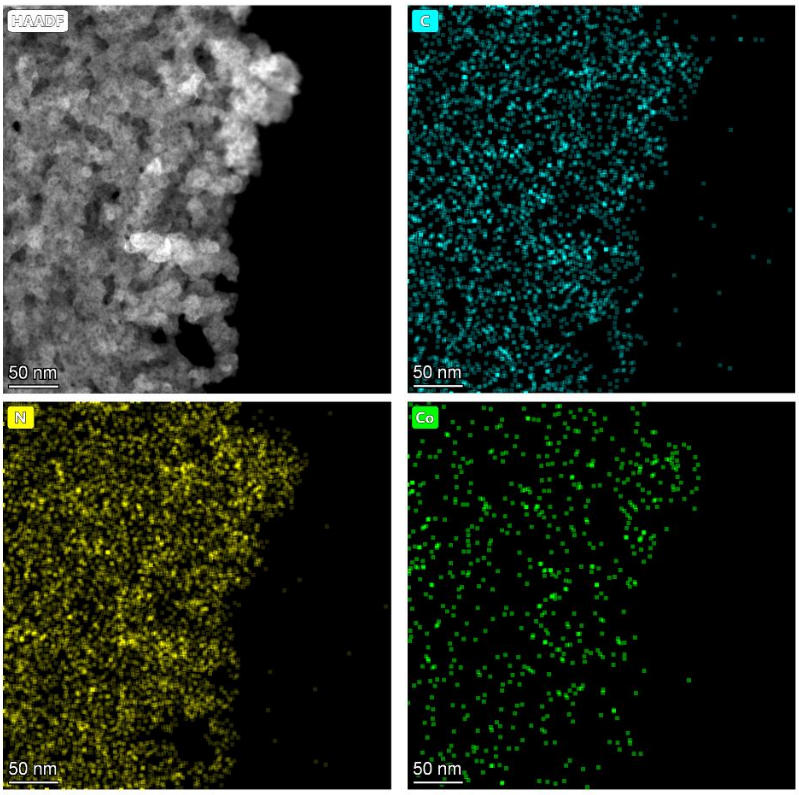

Supplementary Figure 4. EDS mapping of Co_{SAPs}-PuCN.

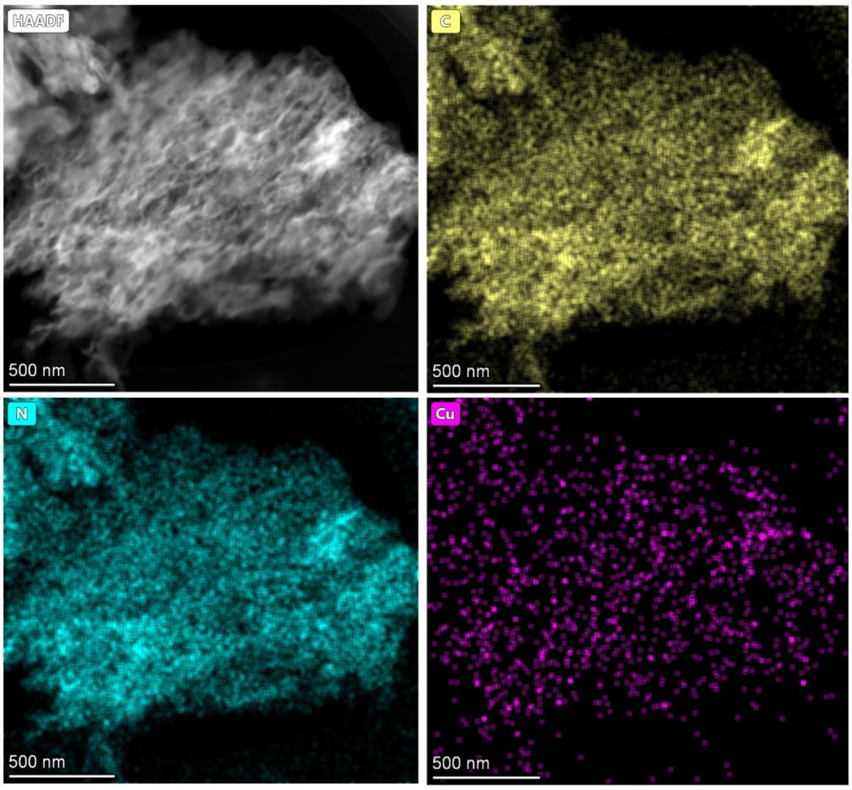

Supplementary Figure 5. EDS mapping of Cu_{SAPs}-PuCN.

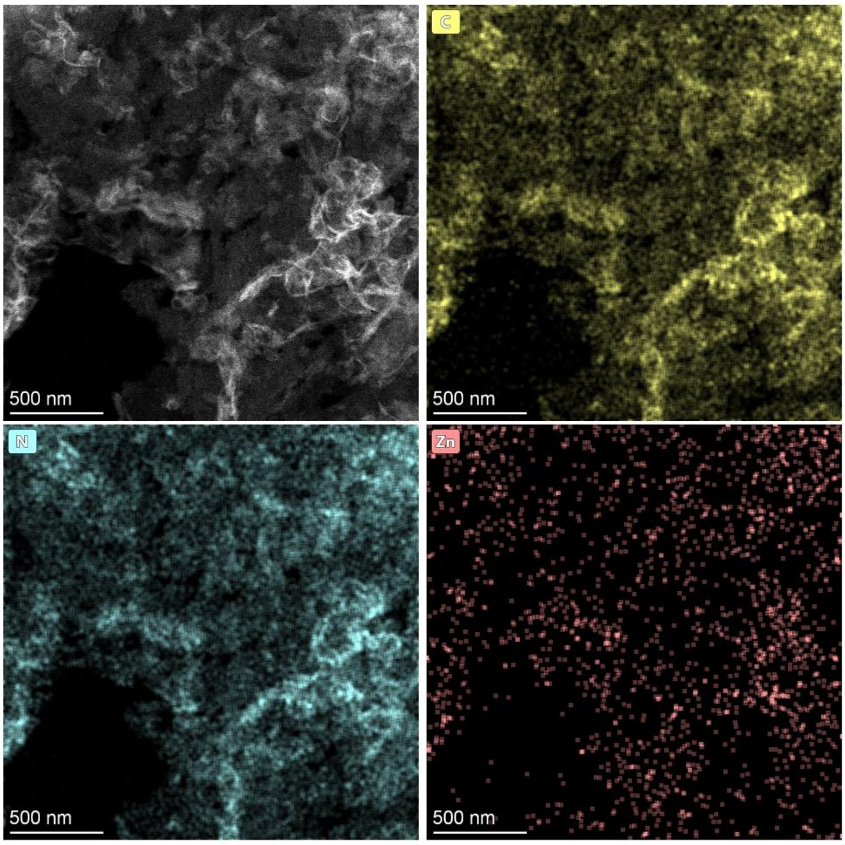

Supplementary Figure 6. EDS mapping of Zn_{SAPs}-PuCN.

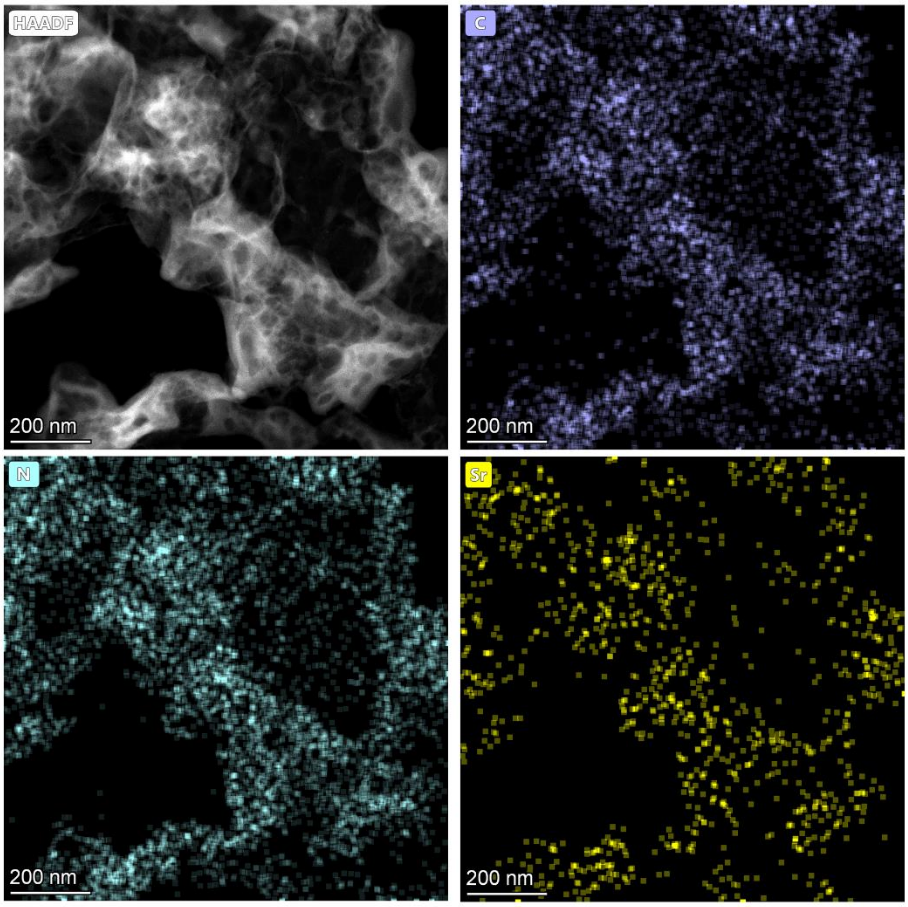

Supplementary Figure 7. EDS mapping of Sr_{SAPs}-PuCN.

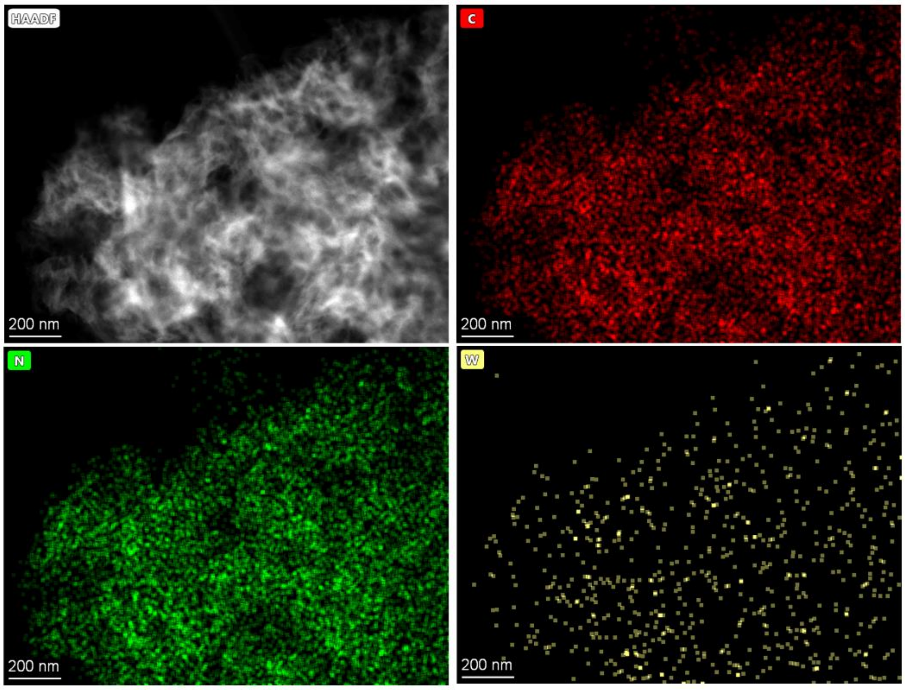

Supplementary Figure 8. EDS mapping of W_{SAPs}-PuCN.

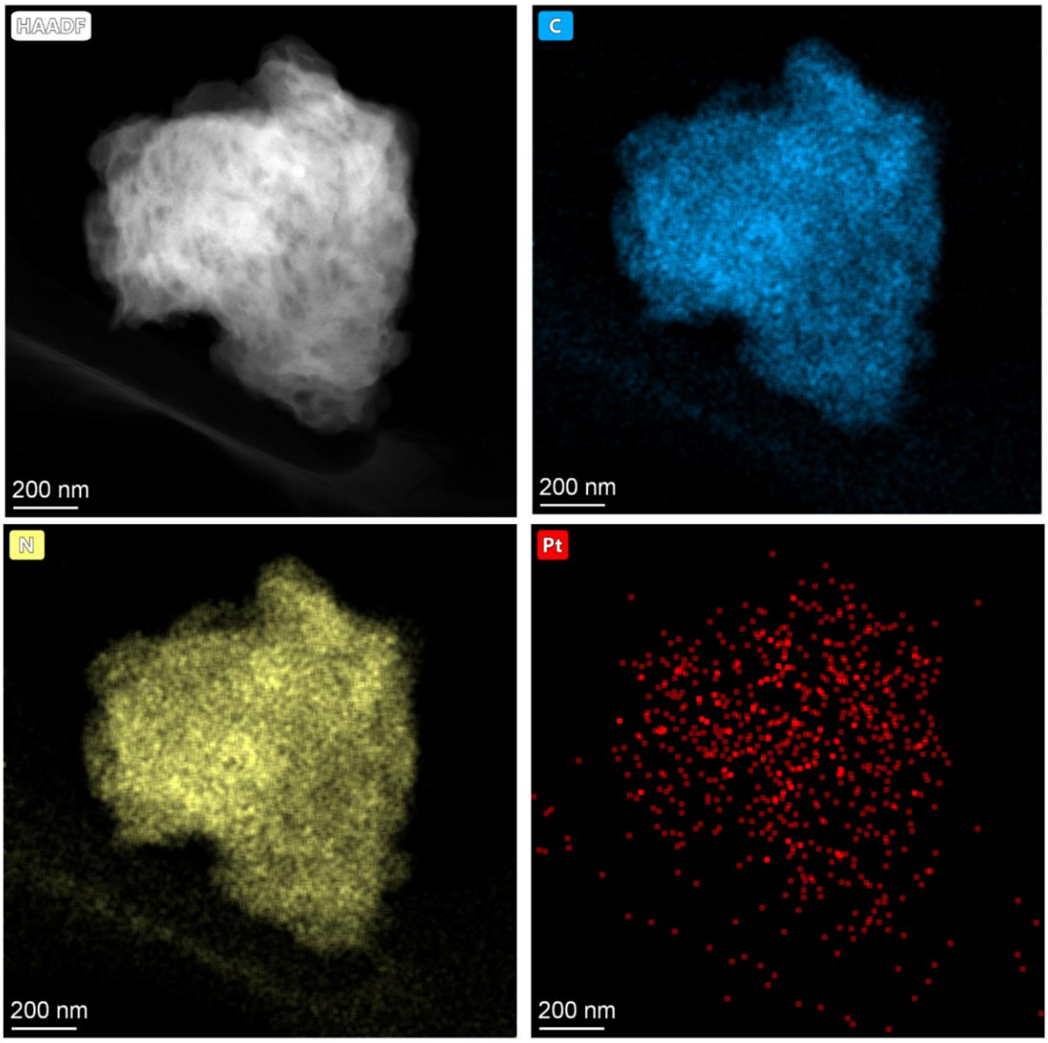

Supplementary Figure 9. EDS mapping of Pt_{SAPs}-PuCN.

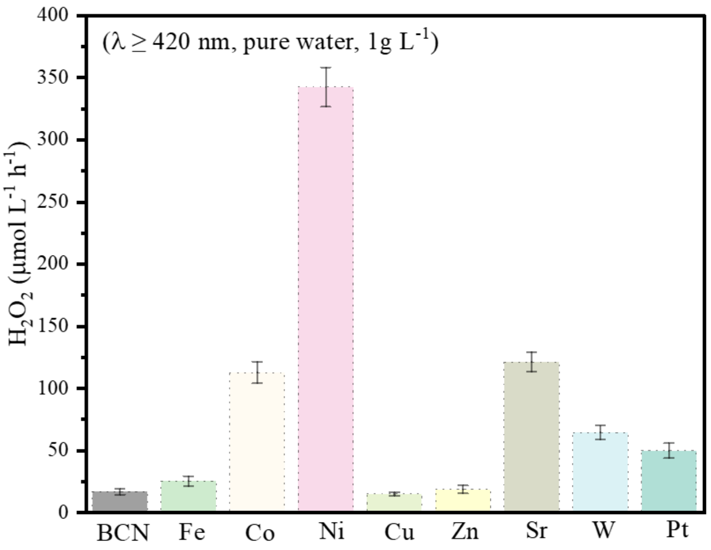

Supplementary Figure 10. Comparison of photocatalytic H_2O_2 generation activity between BCN and prepared high-loading M_{SAPs}-PuCN (M=Fe, Co, Ni, Cu, Zn, Sr, W, Pt). Reaction conditions: Pure water, $\lambda \geq 420$ nm, 60 mW cm^{-2}; 30 mg catalyst in 30 ml pure water, 1 g L^{-1} catalyst; 25 °C. Error bars are the standard deviations of three replicate measurements

As shown in Supplementary Figure 10, in preliminary screening, we found that the introduction of Fe, Cu, and Zn single atoms basically did not improve the activity of H_2O_2 generation, which may be because these metal single atoms are prone to Fenton reaction with H_2O_2 ($M^{n+} + H_2O_2 \rightarrow M^{(n+1)+} + \cdot OH + OH^-$)[1-5]. Compared with the introduction of Co, W, Pt and Sr single atoms, Ni_{SAPs}-PuCN can promote the H_2O_2 formation more obviously, so the structure-activity relationship between Ni_{SAPs}-PuCN and photocatalytic H_2O_2 activity is focused on in this work.

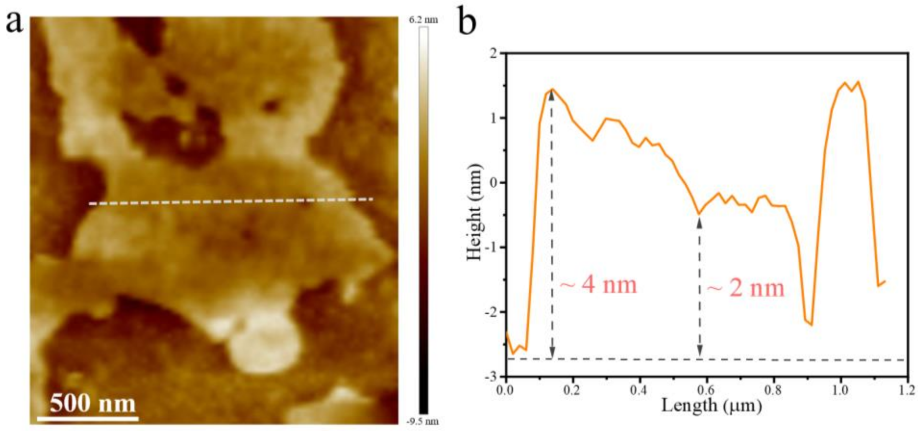

Supplementary Figure 11. (a) AFM image of Ni_{SAPs}-PuCN and (b) the height profile determined along the line in the AFM image.

In **Supplementary Figure 11,** the AFM image also shows that Ni_{SAPs}-PuCN has a porous thin-layer structure, and the thickness of these undulating thin layers is only about 2 ~ 4 nm (about 6 ~ 12 layers).

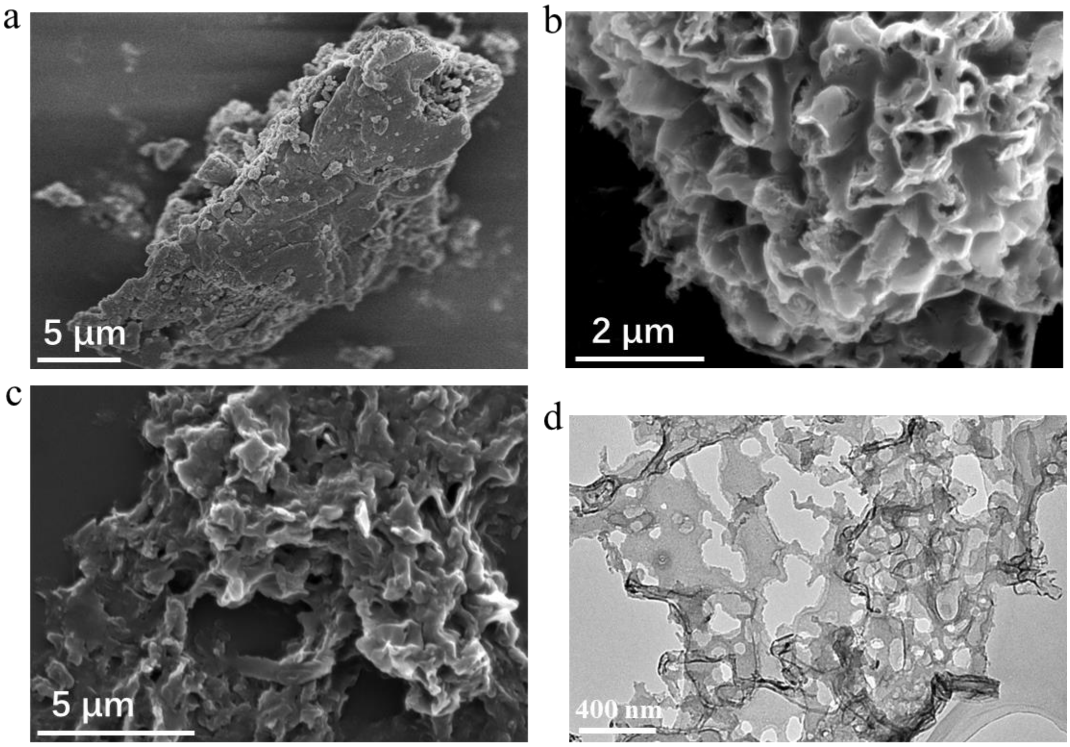

Supplementary Figure 12. SEM images of (a) BCN, (b) PuCN, and (c) Ni_{SAPs}-PuCN. TEM image of (d) Ni_{SAPs}-PuCN.

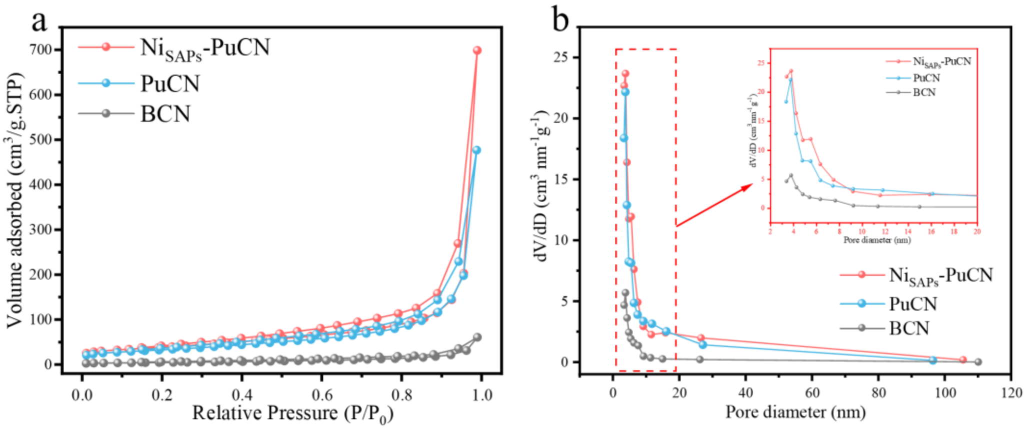

Supplementary Figure 13. BET characterization of all samples. (a) N_2 adsorption-desorption isotherms and (b) pore size distribution curves for BCN, PuCN, and Ni_{SAPs}-PuCN.

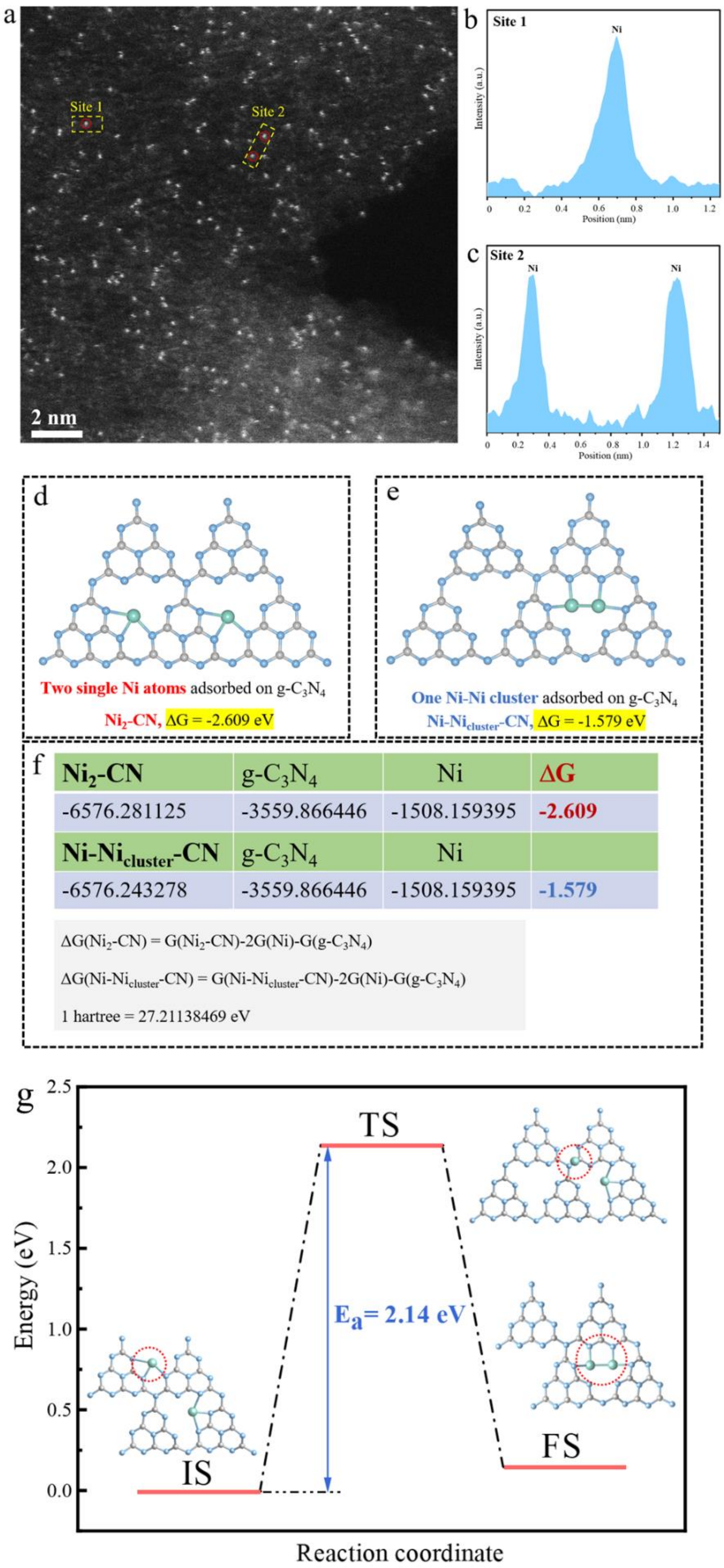

Supplementary Figure 14. Rationality and stability of Ni single atoms on Ni_{SAPs}-PuCN. (a) Aberration-corrected HAADF-STEM image of Ni_{SAPs}-PuCN. (b-c) The intensity profile obtained from the Ni single atom sites (Site 1 and Site 2). The Optimized structures and

corresponding Gibbs free energies of (d) two Ni single atoms and (e) one Ni-Ni cluster adsorbed on g-C_3N_4 at room temperature, where the gray, blue and light green balls are C, N, and Ni atoms, respectively. (f) The original data and calculation formula of this theoretical calculation: The Gibbs free energy (hartree) for related molecules and the ΔG (eV) for two single Ni atoms and one Ni-Ni cluster adsorbed on g-C_3N_4 at room temperature (298.15 K) and 1 atm.

As shown in **Supplementary Figure 14,** we selected typical two site regions (site 1 and site 2) in the aberration-corrected HAADF-STEM image of Ni_{SAPs}-PuCN for intensity analysis. The contrast strength of atoms in aberration-corrected HAADF-STEM images strongly depends on atomic number. In **Supplementary Figure 14b-c**, it can be very intuitively seen that the intensity of single bright spot in the two-site region is significantly higher than that of the base structure (CN structure), which confirms that the Ni single-atom sites are dispersed in g-C_3N_4. The Gibbs free energy for two single Ni atoms (named Ni_2-CN) and one Ni-Ni cluster (named Ni-$Ni_{cluster}$-CN) adsorbed on g-C_3N_4 at room temperature was studied by density functional theory (DFT), where all structures were optimized via Gaussian 16 C.02 at M06-2X functional and the def2-TZVP basis set. Harmonic frequencies were performed at the same level to confirm that the structure corresponds to the minima on the potential energy surfaces, and gained Gibbs free energy at room temperature and 1 atm. The optimized structures of Ni_2-CN and Ni-$Ni_{cluster}$-CN and related Gibbs free energy at room temperature (298.15 K) and 1 atm are shown in **Supplementary Figure 14d-e**. It can be seen that the ΔG (eV) for single Ni atoms adsorbed on g-C_3N_4 is -2.609 eV, indicating that the Ni single atoms are preferred to be anchored on g-C_3N_4. When two Ni atoms (Ni-Ni cluster) are adsorbed on g-C_3N_4, ΔG becomes -1.579 eV (**Supplementary Figure 14f**), indicating that the second Ni atom destabilizes the whole system, which may be that the cavity cannot accommodate two Ni atoms and result in large steric effect. Considering that lower energy corresponds to greater stability, it is evident that the Ni single atom adsorbed on g-C_3N_4 represents the most reasonable and stable structure. Next, as shown in **Supplementary Figure 14g**, we calculated the transition state structure and the activation barriers from the configuration of two Ni single atoms to the Ni-Ni cluster configuration on g-C_3N_4. The results (**Supplementary Figure 14g**) show that this process needs to overcome a large energy barrier (2.14 eV), further indicating that it is difficult for isolated Ni single atoms on g-C_3N_4 to form Ni-Ni clusters.

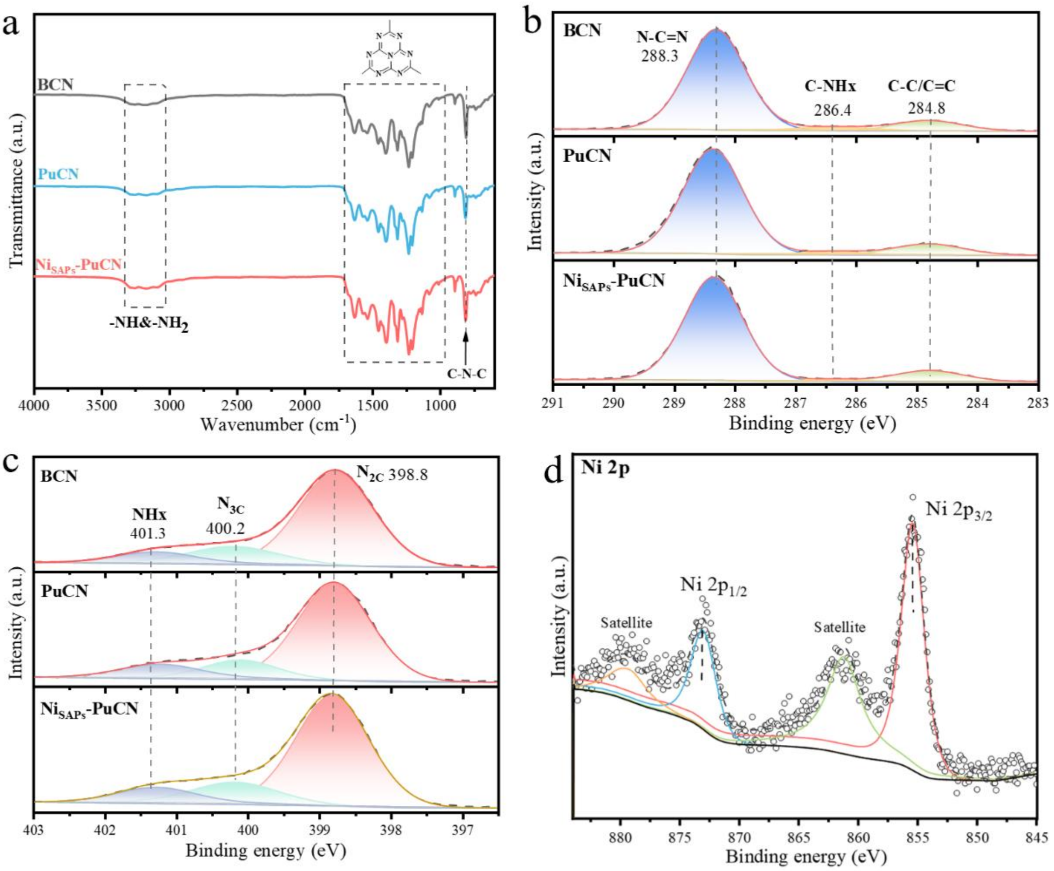

Supplementary Figure 15. Macrostructural information for all samples. (a) FTIR spectra of BCN, PuCN, and Ni$_{SAPs}$-PuCN. (b) C *1s* XPS, (c) N *1s* XPS, and (d) high-resolution Ni *2p* XPS spectra of Ni$_{SAPs}$-PuCN.

In **Supplementary Figure 15a**, the BCN presents a peak at 810 cm^{-1} typical of the out-of-plane bending mode of heptazine rings, and the peaks between 900 and 1800 cm^{-1} are derived from the stretching modes of aromatic C–N heterocycles[6, 7]. Meanwhile, multiple broad peaks in the range of 3000~3500 cm^{-1} originate from N-H stretching vibrations[7]. Compared with BCN, the FTIR spectra of PuCN and Ni$_{SAPs}$-PuCN all exhibit the above structural features, indicating that this synthesis method basically maintains the structure of g-C_3N_4. The structure of the samples was further verified by XPS. The C *1s* XPS spectrum for BCN contained three peaks located at 288.3, 286.4, and 284.8 eV, corresponding to N–C=N in the aromatic rings of the g-C_3N_4 heterocycles, C–NH_X (X = 1, 2), and adventitious carbon, respectively[6-8]. The N *1s* XPS spectra of BCN was composed of three binding peaks at 401.3, 400.2, and 398.8 eV, representing NH_X groups, tri-coordinated N (N_{3C}), and bi-coordinated N (N_{2C}) in the framework, respectively. Compared with BCN, C *1s* and N *1s* of PuCN and Ni$_{SAPs}$-PuCN did not change significantly. Meanwhile, the surface C/N element ratios on the XPS of BCN, PuCN and Ni$_{SAPs}$-PuCN are 0.771, 0.787 and 0.789, respectively, indicating that there are almost no obvious defects in the samples. As shown in **Supplementary Figure 15d**, the high-resolution Ni *2p* spectrum of Ni$_{SAPs}$-PuCN exhibits a typical Ni $2p_{3/2}$ and Ni $2p_{1/2}$ doublet accompanied by two satellite peaks. The Ni $2p_{3/2}$ binding energy in Ni$_{SAPs}$-PuCN (about 855.4 eV) is higher than that of Ni metal (852.6 eV), indicating the positive oxidation state of Ni single atoms[9, 10].

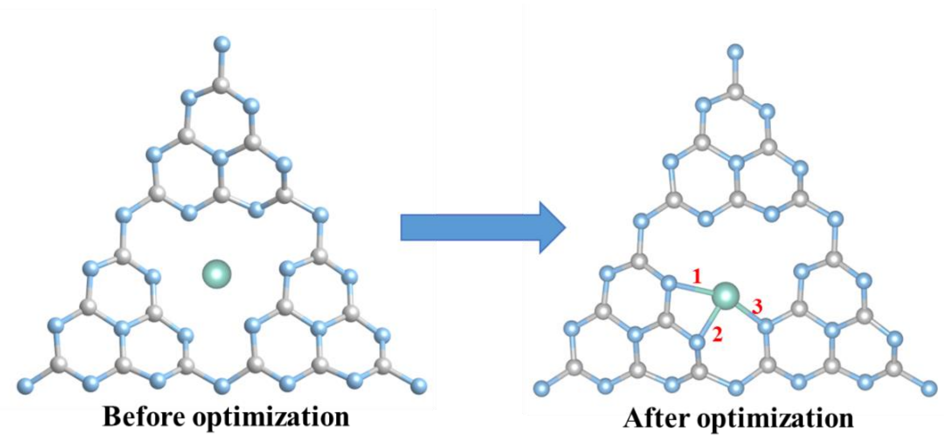

Supplementary Figure 16. Initial and optimized coordination structures of Ni single atoms on g-C_3N_4 structural units (Light green: Ni single atom. Blue: N atoms. Grey: C atoms).

The coordination information of Ni single atoms in g-C_3N_4 was further verified using theoretical calculations. As shown in **Supplementary Figure 16**, the Ni-N_3 coordination can be stably formed after optimization. On one hand, the theoretically calculated coordination number of Ni single atom is consistent with EXAFS. On the other hand, the average Ni-N bond length provided by theoretical calculation is 1.98 Å, which matches the average Ni-N bond length (2.07 Å) measured by XAFS as high as 96%.

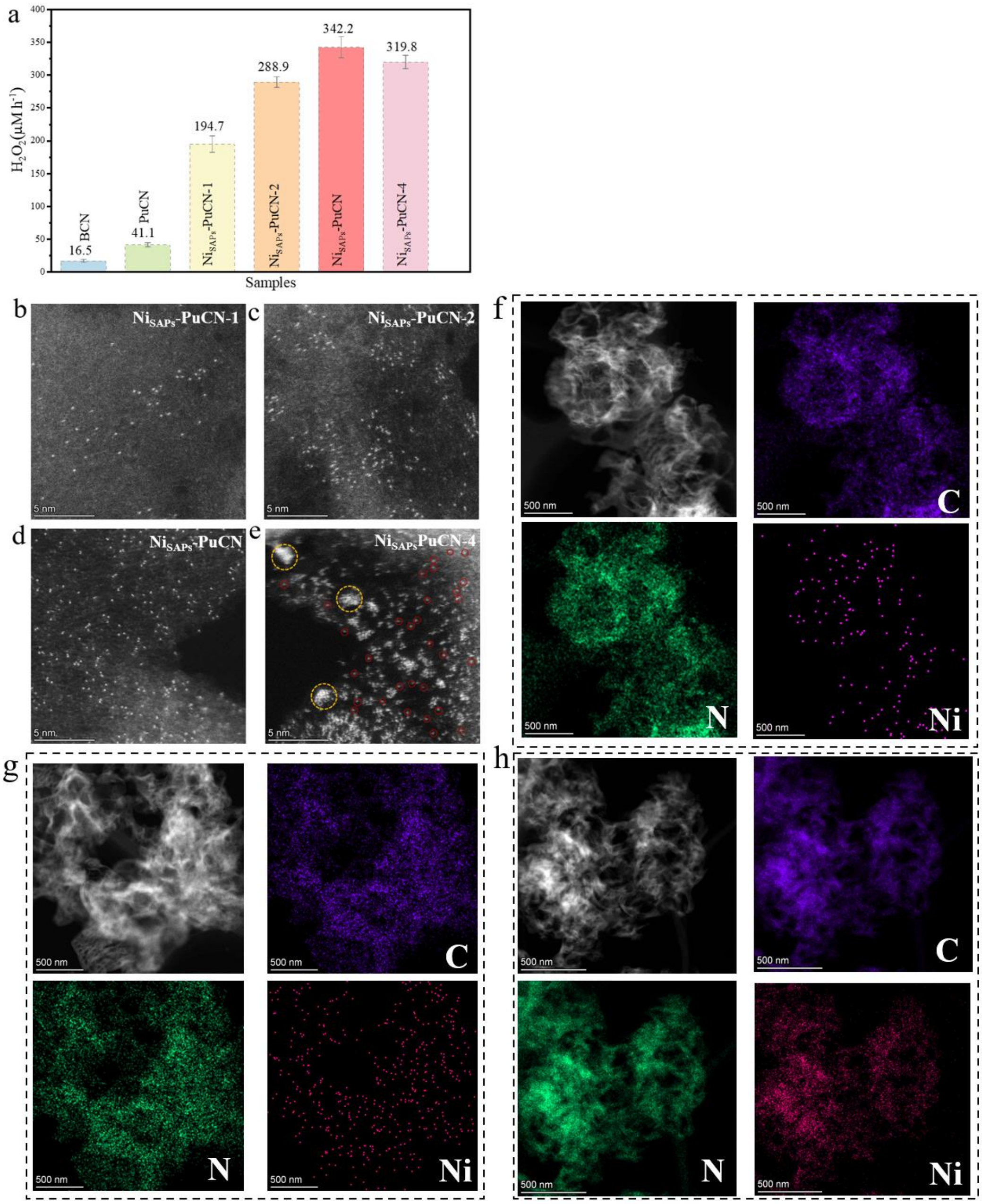

Supplementary Figure 17. Effect of different Ni single atom loading amounts on photocatalytic H_2O_2 activity. (a) Comparison of the photocatalytic H_2O_2 activity of Ni$_{SAPs}$-PuCN with different Ni single atom loadings (Pure water, λ ≥ 420 nm, 60 mW cm^{-2}; 30 mg catalyst in 30 ml pure water, 1 g L^{-1} catalyst; 25 °C). Error bars are the standard deviations of three replicate measurements. (b-e) HAADF-STEM images of different Ni single atom loadings (samples are Ni$_{SAPs}$-PuCN-1, Ni$_{SAPs}$-PuCN-2, Ni$_{SAPs}$-PuCN, Ni$_{SAPs}$-PuCN-4 in turn). The EDS mapping images of (f) Ni$_{SAPs}$-PuCN-1, (g) Ni$_{SAPs}$-PuCN-2, and (h) Ni$_{SAPs}$-PuCN-4.

In **Supplementary Figure 17a,** from Ni$_{SAPs}$-PuCN-1 to Ni$_{SAPs}$-PuCN-2 to Ni$_{SAPs}$-PuCN, the H_2O_2 generation activity gradually increases with the increase of Ni single atom loading in the sample. The Ni contents in Ni$_{SAPs}$-PuCN-1 and Ni$_{SAPs}$-PuCN-2 were 5.1 wt% and 9.5 wt%, respectively (measured by ICP-MS). The highest performing sample is Ni$_{SAPs}$-PuCN with the

Ni content of 12.5 wt%. The HAADF-STEM images of the above three samples are shown in **Supplementary Figure 17b-d.** When the loading of Ni was further increased, the performance of Ni_{SAPs}-PuCN-4 was lower than that of Ni_{SAPs}-PuCN, which was due to the formation of some clusters (about 2 nm) at too high loading (**Supplementary Figure 17e**) and thus reduced catalytic activity (**Supplementary Figure 17a**). In **Supplementary Figure 17f-h**, the EDS images of the samples (Ni_{SAPs}-PuCN-1, Ni_{SAPs}-PuCN-2, and Ni_{SAPs}-PuCN-4) with different Ni single-atom loadings demonstrate the distribution of Ni elements on this porous thin-layer g-C_3N_4 substrate.

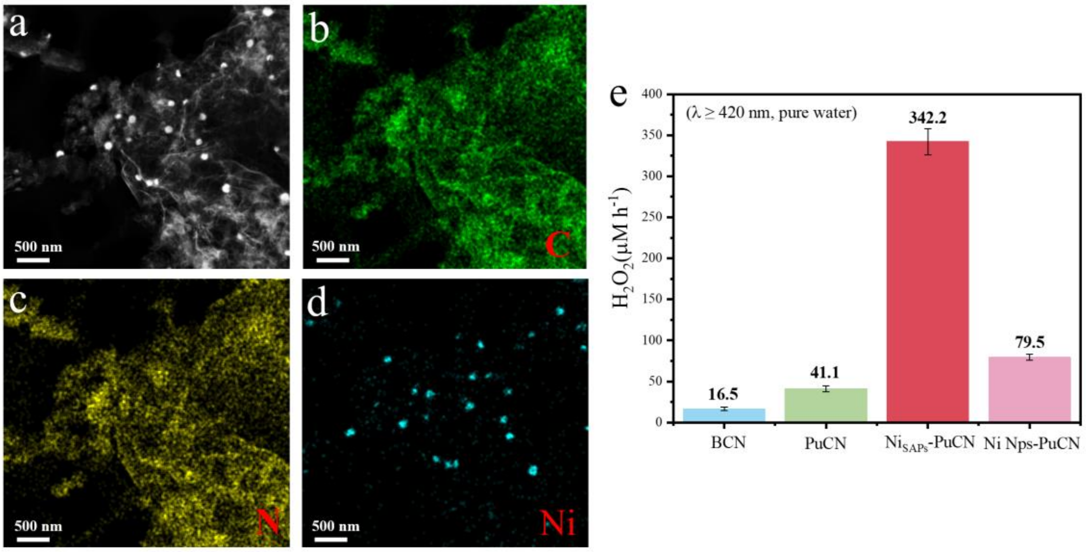

Supplementary Figure 18. Characterization of Ni nanoparticle sample and the effect of Ni nanoparticles on photocatalytic H_2O_2 activity. (a) HAADF-STEM image and (b-d) EDS mapping of Ni Nps-PuCN. (e) Comparison of the photocatalytic H_2O_2 activity of BCN, PuCN, Ni_{SAPs}-PuCN, and Ni Nps-PuCN (Pure water, $\lambda \geq 420$ nm, 60 mW cm^{-2}; 30 mg catalyst in 30 ml pure water, 1 g L^{-1} catalyst; 25 °C). Error bars are the standard deviations of three replicate measurements.

We loaded Ni nanoparticles on PuCN (named Ni Nps-PuCN) using the $NaBH_4$ reduction method. As shown in **Supplementary Figure 18a**, there are abundant Ni nanoparticles on Ni Nps-PuCN, indicating that Ni nanoparticles have been successfully loaded on PuCN. The EDS images (**Supplementary Figure 18b-d**) further show that C and N are uniformly distributed on the Ni Nps-PuCN, and Ni elements are concentrated on the nanoparticles. The photocatalytic H_2O_2 activities of Ni_{SAPs}-PuCN and Ni Nps-PuCN were measured under the same conditions. As shown in Figure **Supplementary Figure 18e**, the H_2O_2 activity of Ni_{SAPs}-PuCN is significantly higher than that of Ni Nps-PuCN, which indicates that Ni single atoms on PuCN have stronger catalytic activity than Ni nanoparticles.

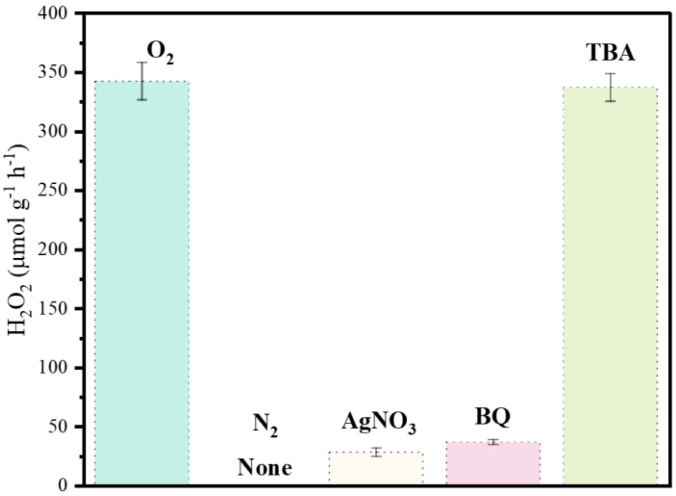

Supplementary Figure 19. The photocatalytic H_2O_2 generation rates of Ni_{SAPs}-PuCN under different reaction gases or different sacrificial agents. Error bars are the standard deviations of three replicate measurements.

In **Supplementary Figure 19**, when the O_2 in the reaction was replaced by N_2, almost no H_2O_2 was detected, indicating that O_2 is a necessary reactant. To further understand the photocatalytic H_2O_2 evolution mechanism over Ni_{SAPs}-PuCN in pure water, $AgNO_3$ and benzoquinone (BQ) were used as electron (e^-) and superoxide radical scavengers to perform active species trapping experiments. When $AgNO_3$ (0.1 mM) was added to the reaction system, the H_2O_2 yield dropped rapidly, which indicated that H_2O_2 was generated by electron reduction of O_2. Notably, when BQ (0.1 mM) was added to the system, the yield of H_2O_2 was very low, suggesting that $\cdot O_2^-$ is a necessary intermediate for the generation of H_2O_2 ($O_2 \rightarrow \cdot O_2^- \rightarrow H_2O_2$). The reaction mechanism was explored using tert-butanol (TBA) as a hydroxyl radical ($\cdot$OH) scavenger. After adding TBA, the H_2O_2 activity of Ni_{SAPs}-PuCN remained basically unchanged, which indicated that hydroxyl radicals did not participate in the generation of H_2O_2.

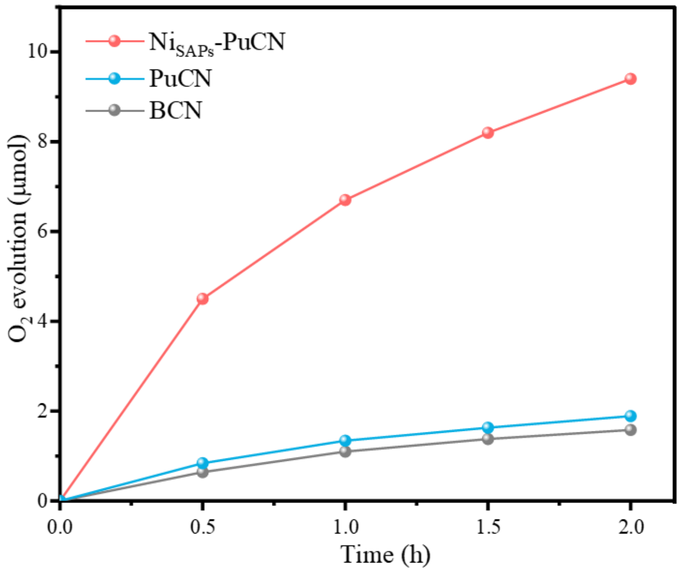

Supplementary Figure 20. Time course of photocatalytic O_2 evolution measured over BCN, PuCN and Ni$_{SAPs}$-PuCN ($\lambda \geq 420$ nm; 1 g L^{-1} La_2O_3 and 20 mM $AgNO_3$; 1 g L^{-1} catalyst, 30 mg catalyst in 30 ml solution).

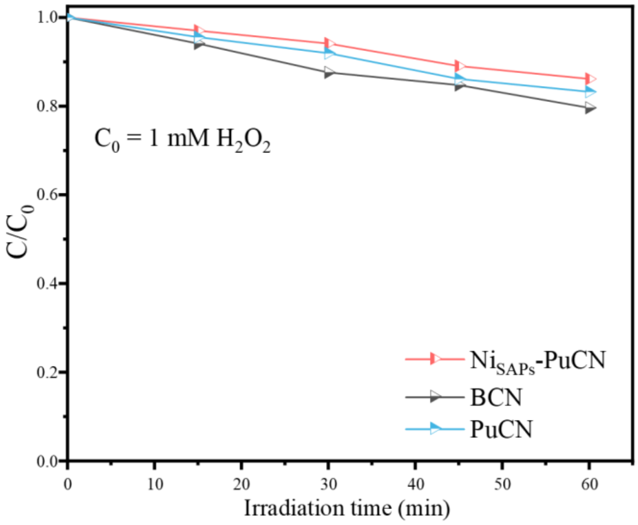

Supplementary Figure 21. Photocatalytic decomposition of H_2O_2 (1 mM) on the samples under visible light irradiation ($\lambda \geq 420$ nm, 60 mW cm^{-2}; 30 mg catalyst in 1 mM H_2O_2 solution).

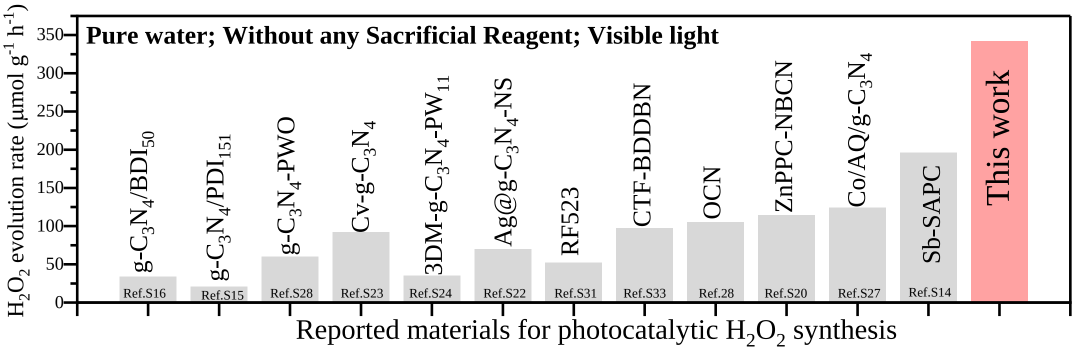

Supplementary Figure 22. In pure water system, a comparison of the H_2O_2 generation performance (μmol g^{-1} h^{-1}) of Ni_{SAPs}-PuCN and other photocatalysts in recent years. The comparison details of all the above samples are collated in **Supplementary Table 4**.

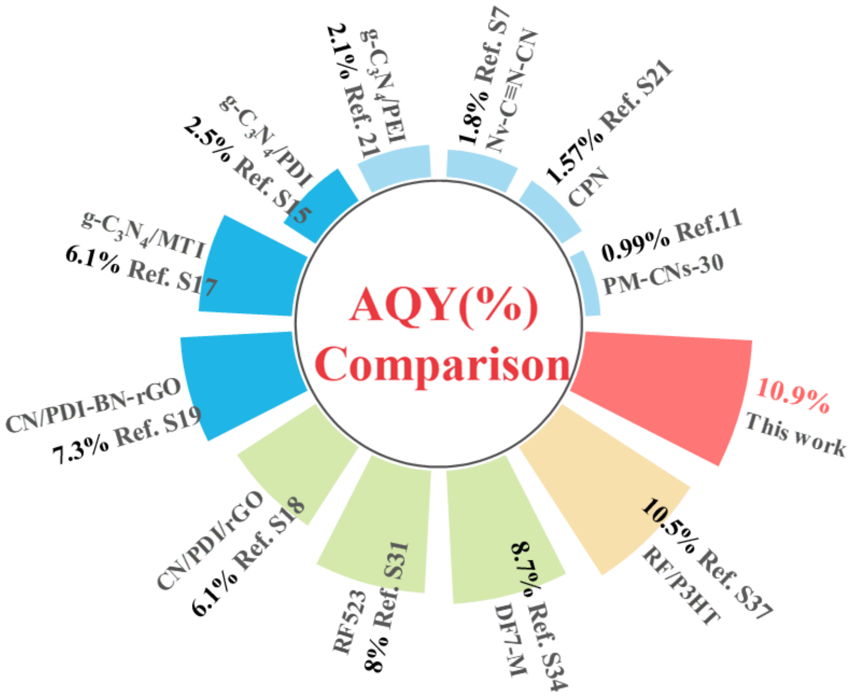

Supplementary Figure 23. Summarized AQY at 420 nm of recently reported photocatalysts (g-C_3N_4-based and other types of photocatalysts) for H_2O_2 production in pure water. The comparison details of all the above samples are collated in **Supplementary Table 4**.

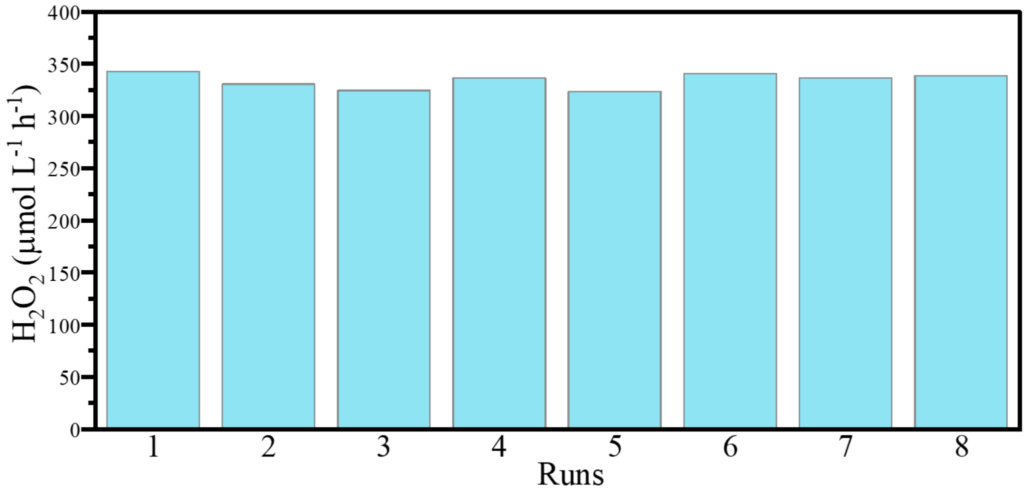

Supplementary Figure 24. Cyclic test of photocatalytic H_2O_2 production over Ni_{SAPs}-PuCN.

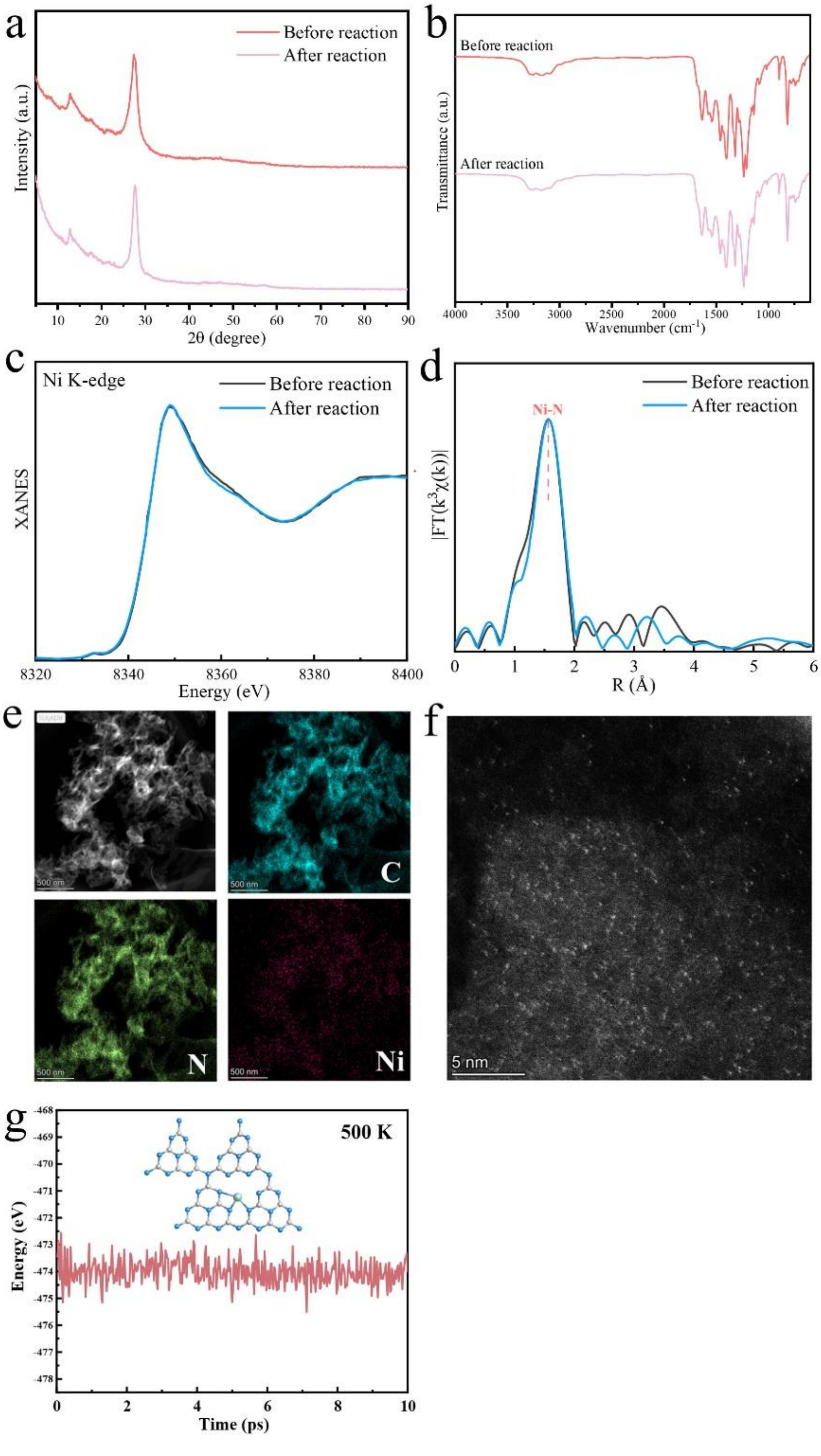

Supplementary Figure 25. Structural stability of Ni_{SAPs}-PuCN after photocatalytic 8-cycle reaction. The (a) XRD and (b) FTIR spectrum of Ni_{SAPs}-PuCN after the photocatalytic reaction. (c) Ni K-edge XANES spectra of the Ni_{SAPs}-PuCN sample before and after the 8 cycles of reaction. (d) Fourier transform of EXAFS spectra of Ni_{SAPs}-PuCN before and after 8 cycles of reaction. (e) EDS mapping and (f) HAADF-STEM image of Ni_{SAPs}-PuCN after 8 cycles of reaction. (g) DFT total energy versus total simulation time at 500 K for the Ni_{SAPs}-PuCN structural model (Ni-N_3). The inset shows the structure at 10 ps.

The XRD and FTIR spectra of Ni_{SAPs}-PuCN **(Supplementary Figure 25a-b)** did not change much before and after the photocatalytic reaction, and the 8-cycle performance did not weaken significantly (**Supplementary Figure 24**), indicating that Ni_{SAPs}-PuCN has good catalytic cycle and stability. Further, the stability of Ni single atoms in Ni_{SAPs}-PuCN after the 8 cycles reaction was confirmed by X-ray absorption fine structure spectroscopy (XAFS). As shown in **Supplementary Figure 25c**, the Ni K-edge X-ray absorption near-edge structure (XANES) spectra of Ni_{SAPs}-PuCN before and after the reaction were basically changed little, proving that the valence state of Ni in Ni_{SAPs}-PuCN was basically unchanged before and after the reaction. Meanwhile, **Supplementary Figure 25d** shows the Fourier transform of the extended X-ray absorption fine structure (FT-EXAFS) spectra of Ni_{SAPs}-PuCN before and after the reaction. It can be seen in **Supplementary Figure 25d** that the Ni_{SAPs}-PuCN after 8 cycles reaction also exhibits a main peak around 1.69 Å (Ni-N). Meanwhile, for the core Ni-N coordination peak, the Ni_{SAPs}-PuCN before and after the cyclic reaction has the same peak intensity and peak position, which indicated that the Ni single-atom coordination structure in Ni_{SAPs}-PuCN did not change after the cyclic reaction. Furthermore, Ni_{SAPs}-PuCN after the cyclic reaction did not detect the appearance of Ni-Ni bond **(Supplementary Figure 25d)**, indicating that there was no agglomeration of Ni single atoms. The EDS mapping (**Supplementary Figure 25e**) showed that the Ni element was still uniformly dispersed in Ni_{SAPs}-PuCN after 8 cycles of reaction. Moreover, the Ni single atoms were still highly dispersed on Ni_{SAPs}-PuCN after the cycling reaction (**Supplementary Figure 25f**), and no Ni nanoclusters or particles were found. In summary, the above characterizations fully proved that Ni single atoms in Ni_{SAPs}-PuCN after long-hour cyclic reaction are stable, indicating the good catalytic cycle and stability of Ni_{SAPs}-PuCN. In addition, in **Supplementary Figure 25g**, the molecular dynamics (MD) calculations performed on the Ni_{SAPs}-PuCN model at a temperature of 500 K are shown (total 10 ps). The fluctuation of the total energy of the Ni_{SAPs}-PuCN model with time evolution is shown in **Supplementary Figure 25g**. It is noteworthy that the energy consistently oscillates in proximity to the equilibrium state throughout the entire simulation, without any observed Ni single-atom coordination change or structural damage, indicating the thermodynamic stability of this Ni-N_3 model.

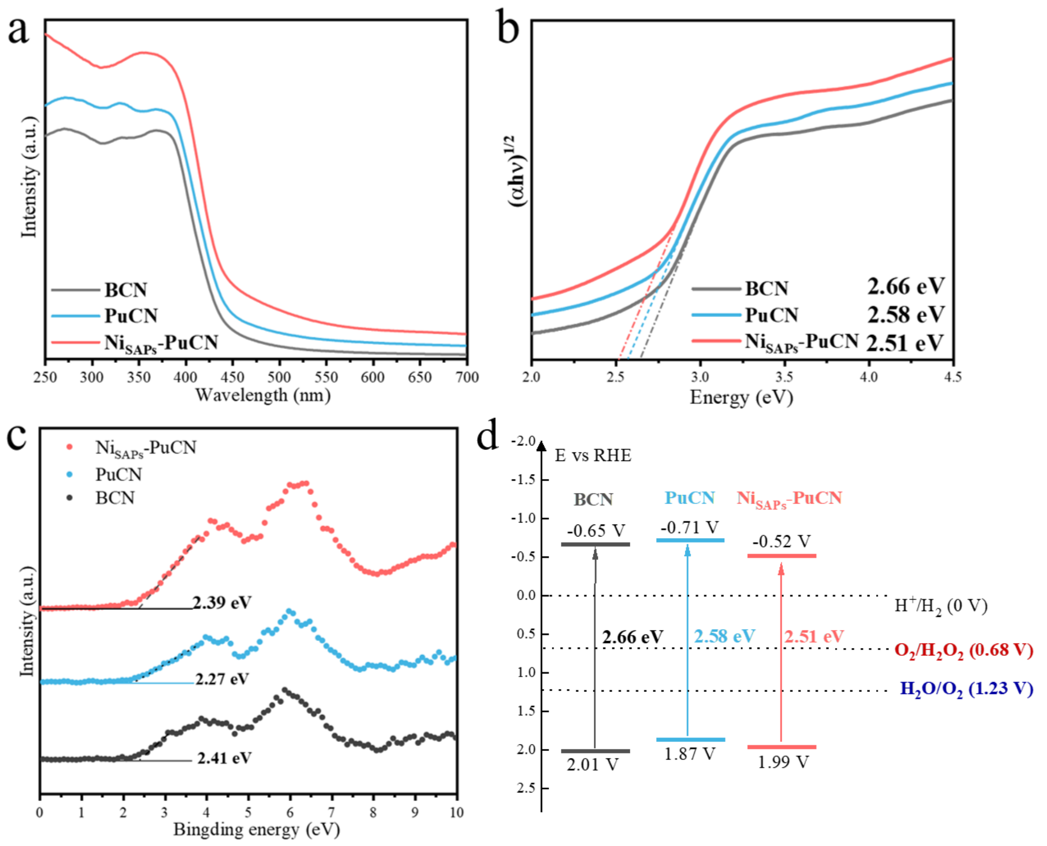

Supplementary Figure 26. Characterization of light absorption properties and band structure of all samples. (a) UV-vis DRS of all samples. (b) Plots of transformed Kubelka-Munk function versus photon energy of all samples. (c) VB XPS spectra of all samples. (d) The schematic diagram of the band gap positions of the samples. The work function of the XPS analyzer is ca. −0.4 eV (vs RHE at pH = 0)[11].

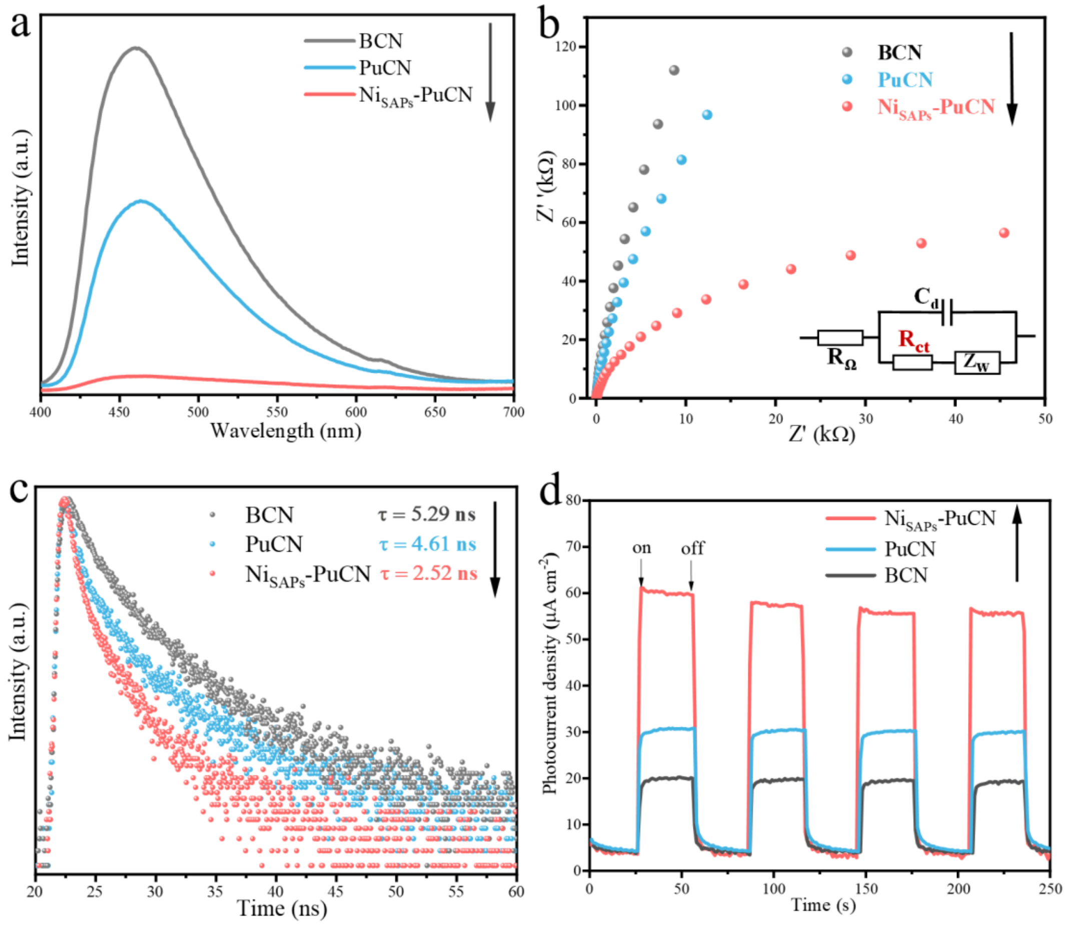

Supplementary Figure 27. Characterization of carrier separation properties of all samples. (a) Photoluminescence (PL) spectra, (b) electrochemical chemical impedance spectra (EIS), (c) time-resolved photoluminescence (TRPL) spectra, and (d) transient photocurrent response (TPR) of BCN, PuCN, and Ni$_{SAPs}$-PuCN.

In Supplementary Figures 27a-b, the Ni$_{SAPS}$-PuCN exhibited the lowest luminescence intensity and the smallest interfacial resistance, which indicated Ni single atoms suppressed the recombination of carriers and facilitated the carrier transport. Meanwhile, TRPL (**Supplementary Fig. 27c**) showed the same trend, in which the average radiation lifetimes of BCN, PuCN and Ni$_{SAPs}$-PuCN were 5.29, 4.61, and 2.52 ns (fitting date in **Supplementary Table 7**) respectively, implying that the Ni-N_3 sites can rapidly trap electrons and accelerate electron transport. In **Supplementary Figure 27d**, compared to BCN and PuCN, Ni$_{SAPs}$-PuCN exhibited a stronger photocurrent density after turning on the light, indicating that the introduction of Ni single atoms facilitated the transport of photogenerated carriers.

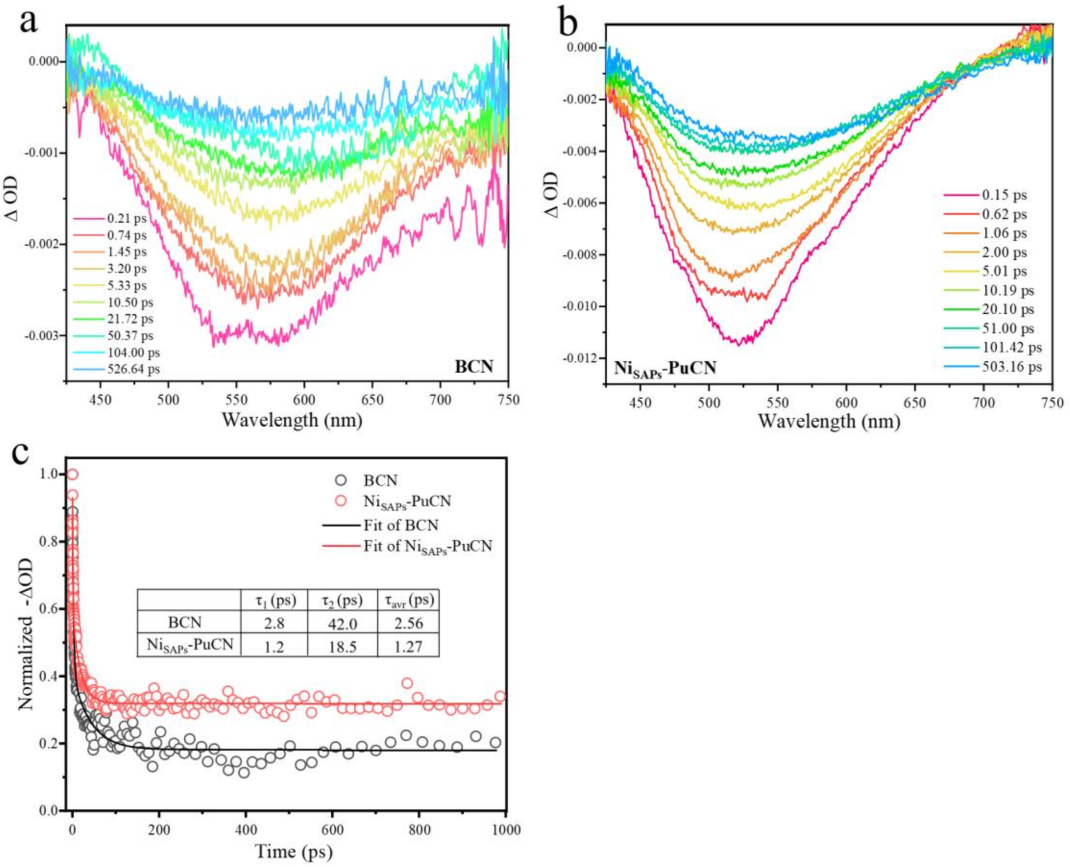

Supplementary Figure 28. Femtosecond transient absorption spectroscopy (fs-TAS) on BCN and Ni$_{SAPs}$-PuCN. Visible transient absorption spectra measurements of (a) BCN and (b) Ni$_{SAPs}$-PuCN with a 400 nm laser flash. (c) Decay kinetics of photogenerated carriers fitted through a double-exponential function (The inset table are fitted lifetimes).

To further monitor the kinetic behaviors of photogenerated carriers, femtosecond transient absorption spectroscopy (fs-TAS) were performed on BCN and Ni$_{SAPs}$-PuCN upon 400 nm laser excitation. The fs-TA data of BCN and Ni$_{SAPs}$-PuCN in water are shown in **Supplementary Figure 28a-b.** The BCN **(Supplementary Figure 28a)** and Ni$_{SAPs}$-PuCN **(Supplementary Figure 28b)** have broad negative signals from 420 to 650 nm, which are attributed to ground state bleaching and stimulated emission[12, 13]. The decay kinetics of the photo-excited carriers of BCN and Ni$_{SAPs}$-PuCN were probing at 540 nm and fitting through a double-exponential function, as shown in **Supplementary Figure 28c**. The τ_1 and τ_2 in **Supplementary Figure 28c** are designated as the shallow trapping of electrons and the recombination of shallowly trapped electrons, respectively. The average lifetimes (τ_{avr}) of BCN and Ni$_{SAPs}$-PuCN after fitting are 2.56 and 1.27 ps, respectively. The slower time scale of intrinsic deep trapped states in BCN corresponds to a longer lifetime, which has been shown to lead to poorer photocatalytic activity[13, 14]. Obviously, due to the introduction of Ni single atoms, Ni$_{SAPs}$-PuCN has a shorter lifetime, which could be attributed to the generated deeply trapped sites, and has been demonstrated to contribute to the 2e$^-$ oxygen reduction reaction (ORR) process[7, 14].

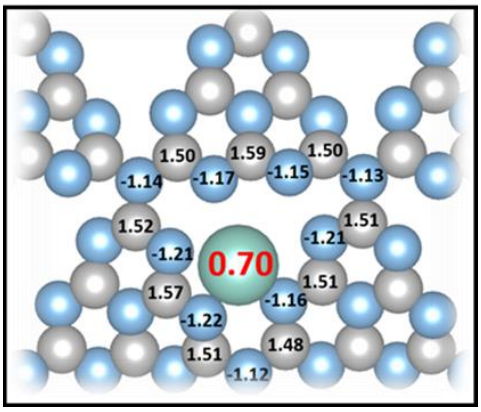

Supplementary Figure 29. Bader charge analysis of Ni_{SAPs}-PuCN (Light green: Ni single atom. Blue: N atoms. Grey: C atoms).

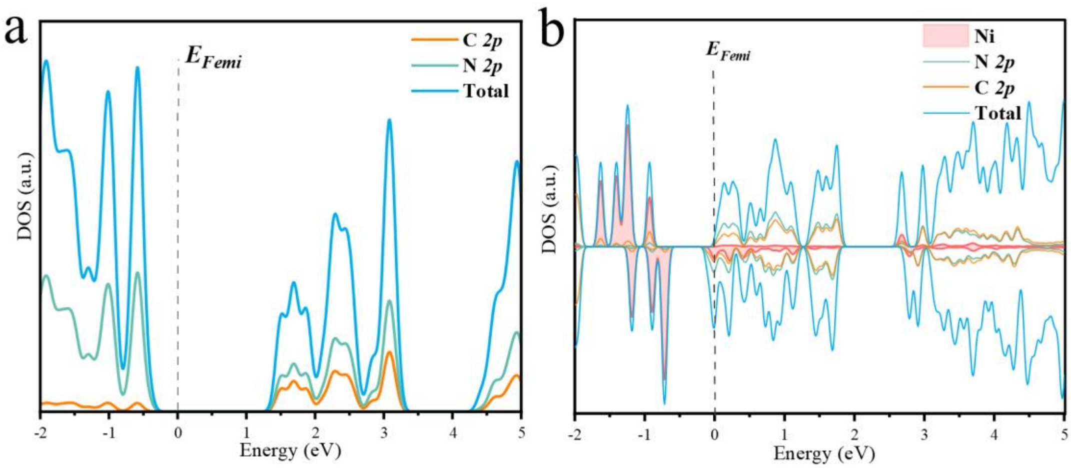

Supplementary Figure 30. TDOS results of (a) BCN and (b) Ni_{SAPs}-PuCN.

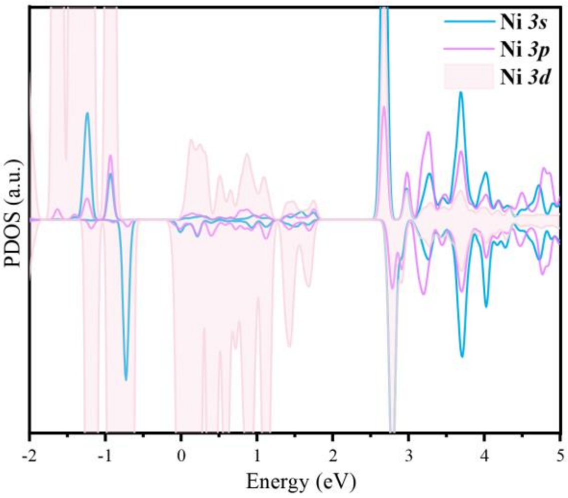

Supplementary Figure 31. PDOS of Ni in Ni_{SAPs}-PuCN.

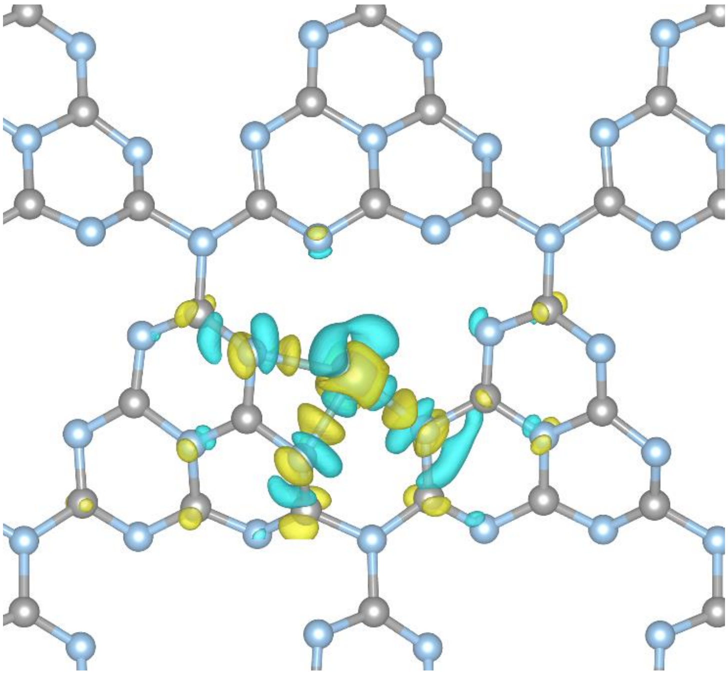

Supplementary Figure 32. Charge density differences (isosurface value 0.005 e Å^{-3}) for Ni$_{SAPs}$-PuCN, in which yellow and cyan represent electron accumulation and depletion, respectively (Light green: Ni single atom. Blue: N atoms. Grey: C atoms).

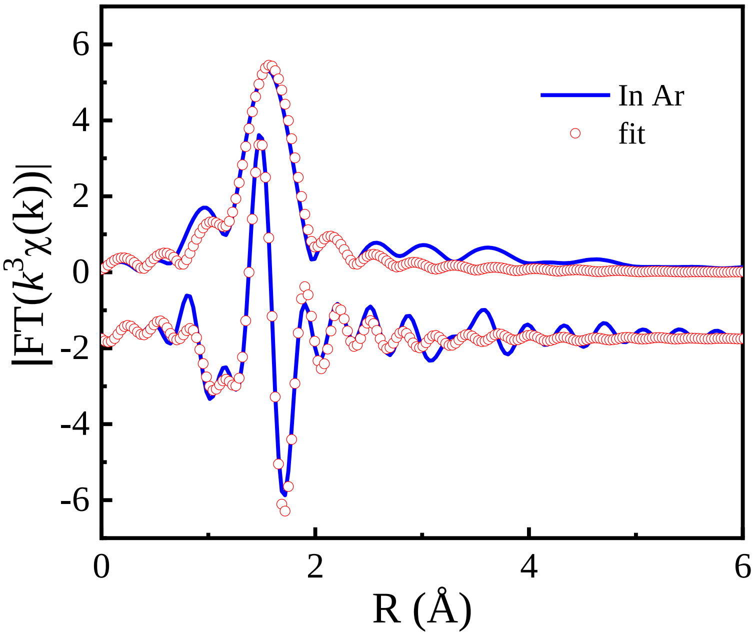

Supplementary Figure 33. First-shell (Ni–N) fitting of Fourier transformations of EXAFS spectra for Ni$_{SAPs}$-PuCN in Ar-saturated aqueous solution.

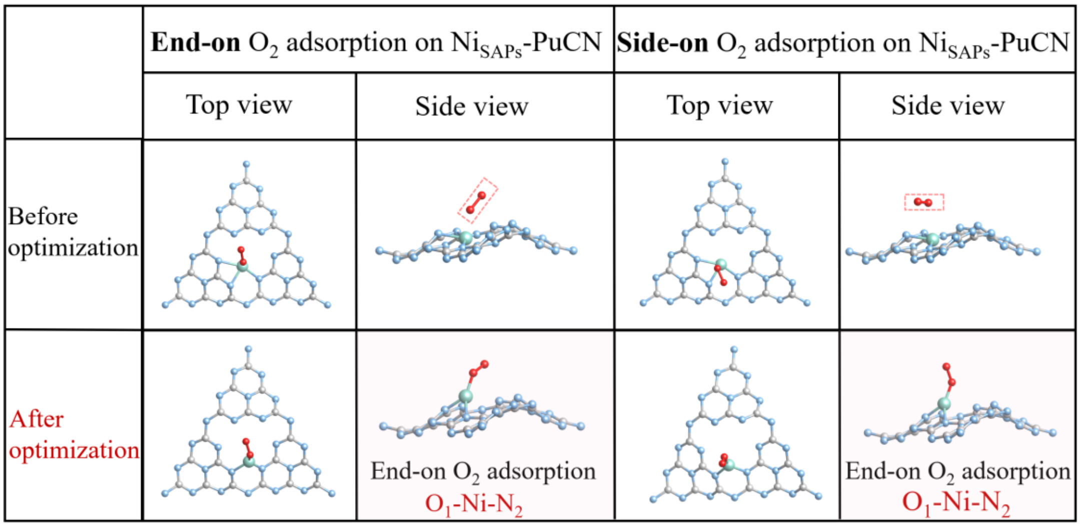

Supplementary Figure 34. Theoretical calculation results of different O_2 adsorption configurations on Ni_{SAPs}-PuCN. The effect of different O_2 adsorption configurations on the Ni_{SAPs}-PuCN structure (before and after optimization) was investigated using theoretical calculations.

As shown in **Supplementary Figure 34,** we provided an end-on O_2 adsorption configuration and a side-on O_2 adsorption configuration on the surface of Ni_{SAPs}-PuCN to explore O_2 adsorption on Ni-N_3 site. The initial model before optimization is shown in the figure above. By the theoretical calculations, the optimized results show that no matter which O_2 adsorption configuration is initially set, the O_2 will eventually be adsorbed on Ni atoms in the end-on adsorption configuration, forming O_1-Ni-N_2 coordination. The appearance of O_1-Ni-N_2 could be understood as the self-optimization process of initial Ni-N_3 sites for more efficient adsorption of O_2, which also strongly supports our fitting results.

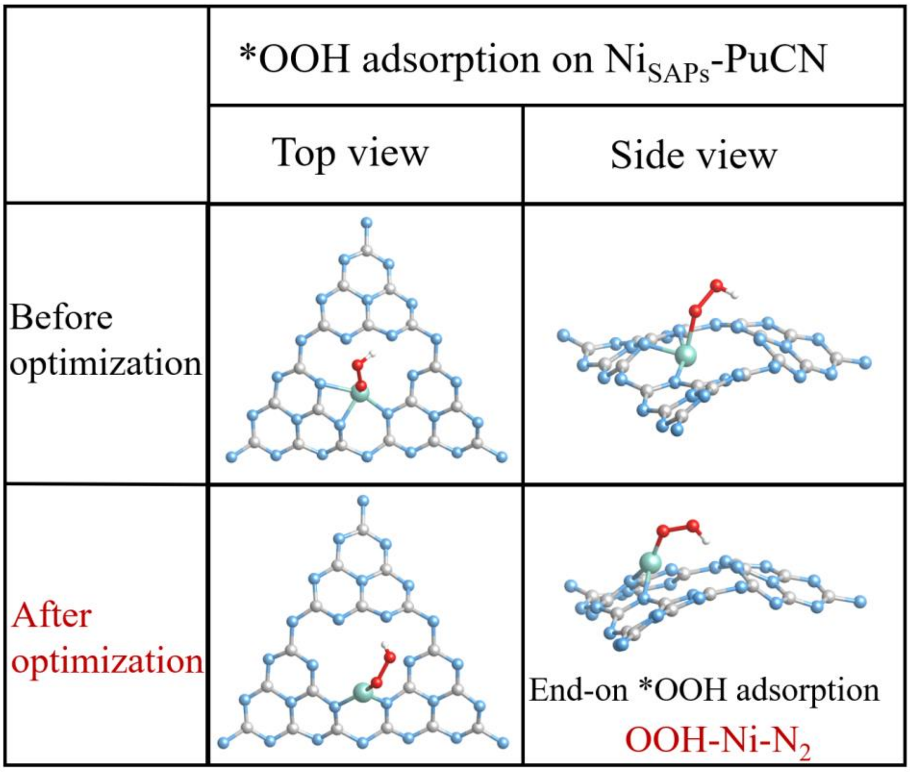

Supplementary Figure 35. Theoretical calculation of *OOH adsorption on Ni$_{SAPs}$-PuCN. The *OOH adsorbed on Ni-N_3 sites in Ni$_{SAPs}$-PuCN structure (before and after optimization) was investigated using theoretical calculations.

As shown in **Supplementary Figure 35,** we first set up Ni-N_3 site in the theoretical calculation system to adsorb *OOH, and the coordination structure of Ni in the initial model is OOH-Ni-N_3 (upper part of **Supplementary Figure 35**). However, the optimized result shows that the coordination structure of Ni is OOH-Ni-N_2, where *OOH is end-on adsorbed on Ni sites (lower part of **Supplementary Figure 35**). This shows that when Ni-N_3 adsorbs and activates O_2, it tends to form end-on adsorbed O_2 (**Supplementary Figure 35**) and further hydrogenates to form end-on *OOH.

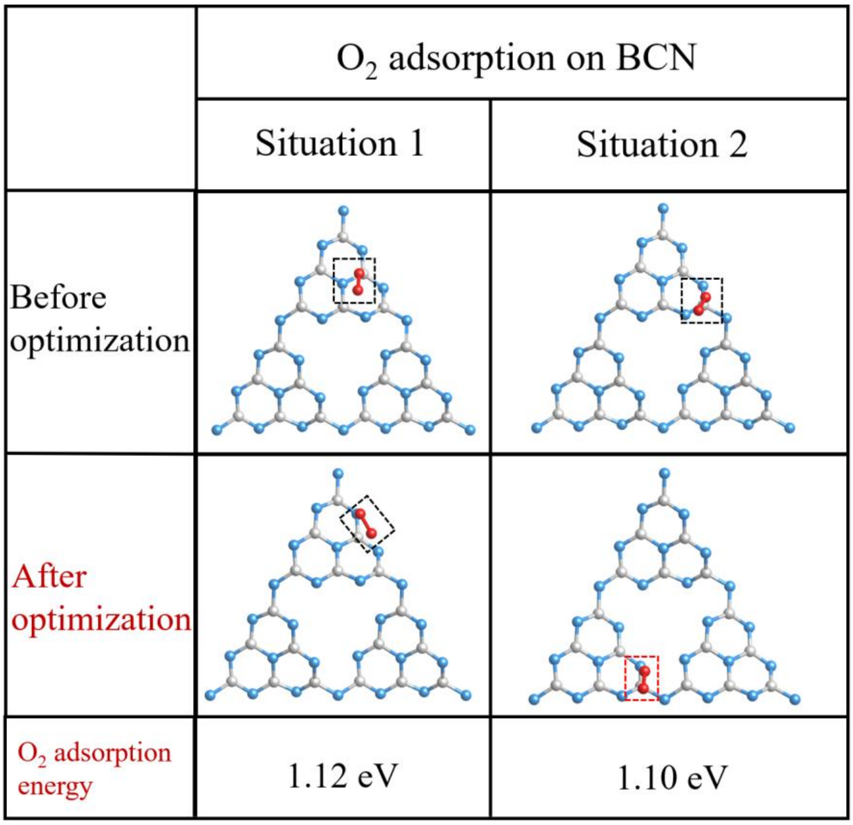

Supplementary Figure 36. Theoretical calculation results of O_2 adsorption on BCN. The adsorption of O_2 on BCN (before and after optimization) was investigated by theoretical calculations.

As shown in **Supplementary Figure 36**, two possible adsorption situations of O_2 on BCN were considered, and the optimized results of theoretical calculations showed the preferred adsorption sites of O_2 on carbon nitride (near N sites). At the same time, the adsorption energy of O_2 in the two cases is positive and the difference is small (1.12 eV and 1.10 eV), indicating that BCN has a weak adsorption on O_2.

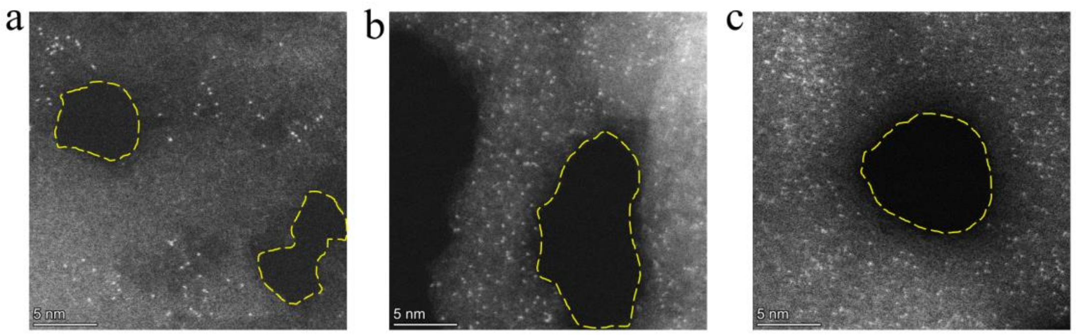

Supplementary Figure 37. Synthesis details of Ni_{SAPs}-PuCN. Aberration-corrected HAADF-STEM image of (a) Ni_{SAPs}-PuCN-1h, (b) Ni_{SAPs}-PuCN-2h, and (c) Ni_{SAPs}-PuCN-3h (the yellow dotted lines represent holes in the sample).

The Ni single-atom samples synthesized under different continuous ultrasonic treatment times (1 h, 2 h, and 3 h) were named Ni_{SAPs}-PuCN-1h, Ni_{SAPs}-PuCN-2h, and Ni_{SAPs}-PuCN-3h in turn. As shown in **Supplementary Figure 37,** with the prolongation of the continuous ultrasonic time (1 h, 2 h, and 3 h), it can be seen intuitively under the spherical aberration transmission electron microscope that Ni single atoms are more uniformly dispersed on the porous thin layer structure and the loading content of Ni single atoms increases significantly. Meanwhile, the inductively coupled plasma mass spectrometry (ICP-MS) further verified that the Ni single atom loadings in Ni_{SAPs}-PuCN-1h, Ni_{SAPs}-PuCN-2h, and Ni_{SAPs}-PuCN-3h were 3.7 wt%, 8.3 wt%, and 12.5 wt%, respectively. Therefore, continuous ultrasonic treatment can effectively promote the uniform distribution of high-loading Ni single atoms on porous ultrathin g-C_3N_4.

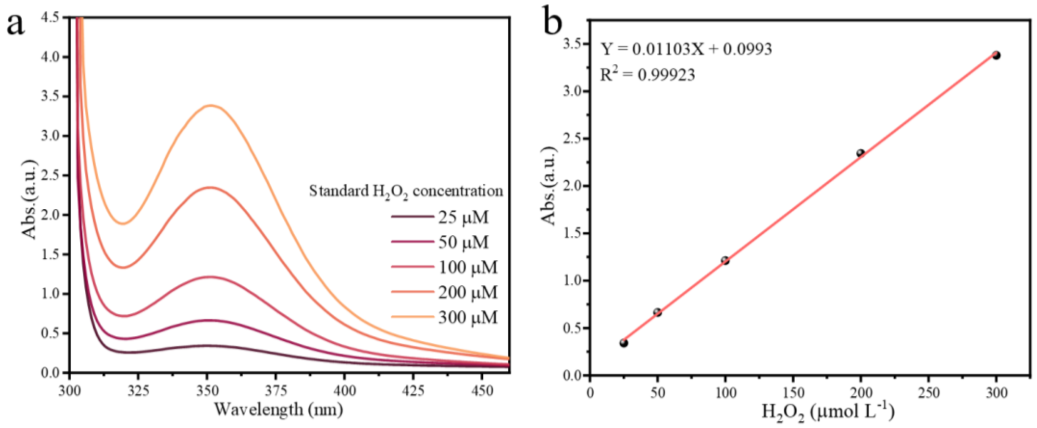

Supplementary Figure 38. Standard curve for H_2O_2 activity testing. (a) Determination of the UV-Vis absorption intensity of different standard concentrations of H_2O_2 by iodometric method. (b) The linear fitting formula for standard H_2O_2 concentration.

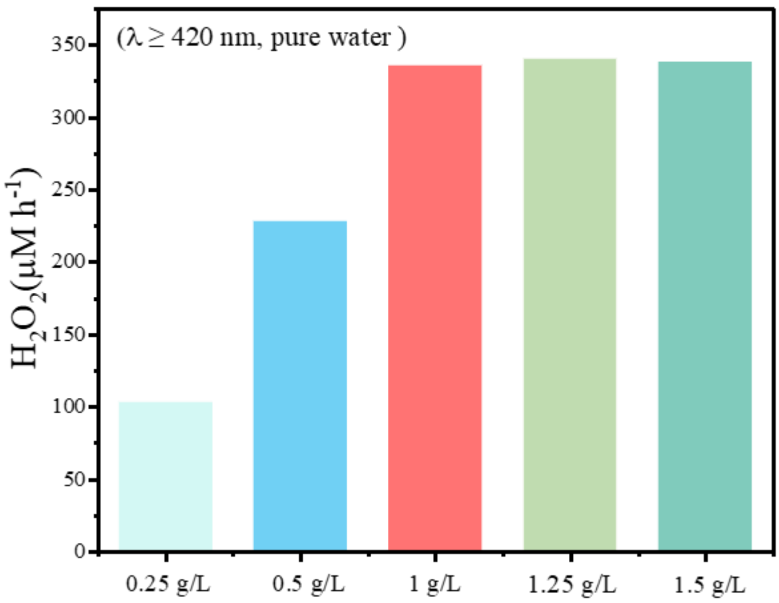

Supplementary Figure 39. The effect of different catalyst concentrations of Ni_{SAPs}-PuCN on the photocatalytic H_2O_2 activity in this work (λ ≥ 420 nm, 60 mW cm^{-2}; 30 ml pure water; 25 ℃).

As shown in **Supplementary Figure 39**, the catalyst concentration to measure the photocatalytic H_2O_2 generation rate (μmol g^{-1} h^{-1}) was confirmed to be 1 g L^{-1} (30 mg in 30 mL) in this work.

Supplementary Table 1. Metal loadings in M_{SAPs}-PuCN (M=Fe, Co, Ni, Cu, Zn, Sr, W, Pt) measured by ICP-MS.

Catalytic	Metal loading (wt%)
Ni_{SAPs}-PuCN	12.5
Fe_{SAPs}-PuCN	11.4
Co_{SAPs}-PuCN	10.3
Cu_{SAPs}-PuCN	11.2
Zn_{SAPs}-PuCN	13.0
Sr_{SAPs}-PuCN	14.7
W_{SAPs}-PuCN	12.8
Pt_{SAPs}-PuCN	14.3

Supplementary Table 2. BET specific surface areas ($m^2\ g^{-1}$) of the samples.

Samples	BCN	PuCN	Ni_{SAPs}-PuCN
BET ($m^2\ g^{-1}$)	16.7	116.1	139.6

Supplementary Table 3. EXAFS fitting parameters at the Ni K-edge for Ni_{SAPs}-PuCN (S_0^2=0.803).

Sample	Shell	N^a	R(Å)^b	σ^2(Å^2)^c	ΔE_0(eV)^d	R factor
Ni_{SAPs}-PuCN	Ni-N	3.2	2.07	0.0055	-1.8	0.0003

$^a N$: coordination numbers; $^b R$: bond distance; $^c\sigma^2$: Debye-Waller factors; $^d\Delta E_0$: the inner potential correction. R factor: goodness of fit. S_0^2 was set to 0.803 for Ni, according to the experimental EXAFS fit of Ni foil reference by fixing CN as the known crystallographic value.

Supplementary Table 4. Performance comparison of recently reported materials for photocatalytic production of H_2O_2 in pure water. The materials of the blue background are g-C_3N_4-based photocatalyst, and the materials of the yellow background are other types of photocatalysts. The standardized yield of H_2O_2 (μmol g^{-1} h^{-1}), AQY, and SCC efficiency are used as evaluation indicators.

catalyst	Reaction solution and catalytic concentration	Light Source	H_2O_2 (μmol g^{-1} h^{-1})	AQY at 420 nm	SCC	Ref.
**Ni_{SAPs}-PuCN**	Pure water (1g L^{-1})	λ ≥ 420 nm	**342.2**	**10.9%**	**1.17% (1h) 0.90% (2h) 0.82% (3h)**	**This work**
g-C_3N_4/PDI	Pure water (1.66 g L^{-1})	λ ≥ 420 nm	21	2.5%	0.1% (2h)	15
g-C_3N_4/BDI	Pure water (1.66 g L^{-1})	λ ≥ 420 nm	34	4.6%	0.13% (2h)	16
g-C_3N_4/MTI	Pure water (1.66 g L^{-1})	λ ≥ 420 nm	22	6.1%	0.18% (2h)	17
g-C_3N_4/PDI/rGO	Pure water (1.66 g L^{-1})	λ ≥ 420 nm	24.1	6.1%	0.2% (2h)	18
g-C_3N_4/PDI-BN-rGO	Pure water (1.66 g L^{-1})	λ ≥ 420 nm	29.1	7.3%	0.28% (2h)	19
Nv-C≡N-CN	Pure water (1 g L^{-1})	λ ≥ 420 nm	137	1.8%	0.23% (1h)	7
ZnPPC-NBCN	Pure water (0.4 g L^{-1})	λ ≥ 420 nm	114	N.T.	N.T.	20
CPN	Pure water (0.66)	λ ≥ 420 nm	246	1.57%	0.43% (8h)	21
Ag@U-g-C_3N_4	Pure water (1 g L^{-1})	λ ≥ 420 nm	70	N.T.	N.T.	22
Cv-g-C_3N_4	Pure water (1 g L^{-1})	λ ≥ 420 nm	90	N.T.	N.T.	23
3DM-g-C_3N_4- PW	Pure water (1 g L^{-1})	λ ≥ 350 nm	35	N.T.	N.T.	24
HJ-C_3N_4	Pure water (1 g L^{-1})	λ ≥ 420 nm	115	N.T.	N.T.	25
K-g-C_3N_4-NH-CH_2-OH	Pure water (1 g L^{-1})	λ ≥ 420 nm	30.4	N.T.	0.29% (1h)	26
Co/AQ/C_3N_4	Pure water (0.5 g L^{-1})	AM 1.5G	124	0.054%	0.014%(1h)	27
G-CN-PWO	Pure water (1 g L^{-1})	λ ≥ 420 nm	60	N.T.	N.T.	28
5Cv@g-C_3N_4	Pure water (1 g L^{-1})	λ ≥ 420 nm	124.5	N.T.	N.T.	29
R370-CN	Pure water (1 g L^{-1})	λ ≥ 420 nm	170	4.3%	0.26% (1h)	30
Sb-SACS	Pure water (2 g L^{-1})	λ ≥ 420 nm	196	17.6%	0.61% (4h)	14
RF523	Pure water (1.66 g L^{-1})	λ ≥ 420 nm	52	8%	0.5% (5h)	31
C_2N_5	Pure water (10 g L^{-1})	λ ≥ 420 nm	155	15.4%	0.55% (6h)	32
CTF-BDDBN	Pure water (0.6 g L^{-1})	λ ≥ 420 nm	97	N.T.	0.14% (1h)	33
DE7-M	Pure water (1.66 g L^{-1})	λ ≥ 420 nm	221.6	8.7%	0.23% (5h)	34
CHF-DPDA	Pure water (2 g L^{-1})	λ ≥ 420 nm	256	16%	0.78% (1h)	35
RF-DHAQ-2	Pure water (0.2 g L^{-1})	λ ≥ 420 nm	1820	11.6%	1.2% (1h)	36
RF/P3HT-1.0	Pure water (1.66 g L^{-1})	λ ≥ 420 nm	123	10.5%	1.0% (3h)	37

Supplementary Table 5. The wavelength-dependent AQY for photocatalytic H_2O_2 generation by Ni_{SAPs}-PuCN in pure water. Conditions: 50 mg catalyst in 50 ml pure water, 25 °C.

Wavelength (nm)	400	420	450	500	550
H_2O_2 (μmol)	8.62	7.21	3.12	0.86	0.32
Light intensity (mW)	10	10.5	10.7	11.0	11.6
Irradiation area (cm^2)	1.69	1.69	1.69	1.69	1.69
Irradiation time (1h)	1	1	1	1	1
AQY (%)	14.3	10.9	4.31	1.04	0.34

λ = 420 nm:

The number of incident photons: $N = \frac{E\lambda}{hv} = \frac{10.5\times10^{-3}\times3600\times420\times10^{-9}}{6.626\times10^{-34}\times3\times10^{8}} = 7.98672\times10^{19}$

$$AQY = \frac{2\times H_2O_2 \text{ formed (mol)}}{\text{the number of incident photons (mol)}} \times 100\% = \frac{2\times7.21\times10^{-6}\times6.02\times10^{23}}{7.98672\times10^{19}} \times 100\% = 10.9\%$$

Supplementary Table 6. EXAFS fitting parameters of Ni_{SAPs}-PuCN in Ar-saturated aqueous solution and O_2-saturated aqueous solution.

Sample	Shell	N^a	R(Å)^b	σ^2(Å^2)^c	ΔE_0(eV)^d	R factor
Ni_{SAPs}-PuCN in Ar-saturated solution	Ni-N	3.1	2.04	0.0033	-3.7	0.004
Ni_{SAPs}-PuCN in O_2-saturated solution	Ni-N	2.1	2.05	0.0031	-2.72	0.003
	Ni-O	1.1	2.10	0.0031	-2.72	0.003

$^a N$: coordination numbers; $^b R$: bond distance; $^c \sigma^2$: Debye-Waller factors; $^d \Delta E_0$: the inner potential correction. R factor: goodness of fit.

Supplementary Table 7. TRPL fitting date of the samples.

Sample	τ_1/ns (A_1)	τ_1/ns (A_2)	τ_A/ns
BCN	2.14 (44.23%)	7.78 (55.77%)	5.29 ns
PuCN	1.21 (42.02%)	7.07 (57.98%)	4.61 ns
Ni_{SAPs}-PuCN	0.79 (47.75%)	4.16 (52.25%)	2.52 ns

The calculation formula of the average TRPL lifetime is: $\tau_A=\frac{\sum Ai\tau i}{\sum Ai}$, where τ_i is the time coefficient and A_i is the corresponding amplitude of each component.

References

1. Xu J*, et al.* Organic wastewater treatment by a single-atom catalyst and electrolytically produced H_2O_2. *Nat Sustain* **4**, 233-241 (2021).
2. Wang B*, et al.* A Site Distance Effect Induced by Reactant Molecule Matchup in Single-Atom Catalysts for Fenton-Like Reactions. *Angew Chem Int Ed Engl* **61**, e202207268 (2022).
3. Zuo S*, et al.* Sandwich structure stabilized atomic Fe catalyst for highly efficient Fenton-like reaction at all pH values. **282**, 119551 (2021).
4. Su L, Wang P, Ma X, Wang J, Zhan S. Regulating Local Electron Density of Iron Single Sites by Introducing Nitrogen Vacancies for Efficient Photo-Fenton Process. *Angew Chem Int Ed Engl* **60**, 21261-21266 (2021).
5. Lin J*, et al.* Functional Carbon Nitride Materials in Photo-Fenton-Like Catalysis for Environmental Remediation. *Advanced Functional Materials* **32**, (2022).
6. Yu H*, et al.* Alkali-Assisted Synthesis of Nitrogen Deficient Graphitic Carbon Nitride with Tunable Band Structures for Efficient Visible-Light-Driven Hydrogen Evolution. *Adv Mater* **29**, (2017).
7. Zhang X*, et al.* Unraveling the dual defect sites in graphite carbon nitride for ultra-high photocatalytic H_2O_2 evolution. *Energy & Environmental Science* **15**, 830-842 (2022).
8. Zhao D*, et al.* Synergy of Dopants and Defects in Graphitic Carbon Nitride with Exceptionally Modulated Band Structures for Efficient Photocatalytic Oxygen Evolution. *Adv Mater* **31**, e1903545 (2019).
9. Zheng T*, et al.* Large-Scale and Highly Selective CO_2 Electrocatalytic Reduction on Nickel Single-Atom Catalyst. *Joule* **3**, 265-278 (2019).
10. Wu Z-Y*, et al.* A general synthesis of single atom catalysts with controllable atomic and mesoporous structures. *Nature Synthesis* **1**, 658-667 (2022).
11. Kong D*, et al.* Tunable Covalent Triazine-Based Frameworks (CTF-0) for Visible-Light-Driven Hydrogen and Oxygen Generation from Water Splitting. *ACS Catal* **9**, 7697-7707 (2019).
12. Jing L, Zhu R, Phillips DL, Yu JC. Effective Prevention of Charge Trapping in

Graphitic Carbon Nitride with Nanosized Red Phosphorus Modification for Superior Photo(electro)catalysis. *Advanced Functional Materials* **27**, (2017).
13. Wang W*, et al.* Femtosecond time-resolved spectroscopic observation of long-lived charge separation in bimetallic sulfide/g-C_3N_4 for boosting photocatalytic H2 evolution. *Applied Catalysis B: Environmental* **282**, (2021).
14. Teng Z*, et al.* Atomically dispersed antimony on carbon nitride for the artificial photosynthesis of hydrogen peroxide. *Nature Catalysis* **4**, 374-384 (2021).
15. Shiraishi Y*, et al.* Sunlight-driven hydrogen peroxide production from water and molecular oxygen by metal-free photocatalysts. *Angew Chem Int Ed Engl* **53**, 13454-13459 (2014).
16. Kofuji Y*, et al.* Graphitic Carbon Nitride Doped with Biphenyl Diimide: Efficient Photocatalyst for Hydrogen Peroxide Production from Water and Molecular Oxygen by Sunlight. *ACS Catalysis* **6**, 7021-7029 (2016).
17. Kofuji Y*, et al.* Mellitic Triimide-Doped Carbon Nitride as Sunlight-Driven Photocatalysts for Hydrogen Peroxide Production. *ACS Sustainable Chemistry & Engineering* **5**, 6478-6485 (2017).
18. Kofuji Y*, et al.* Carbon Nitride-Aromatic Diimide-Graphene Nanohybrids: Metal-Free Photocatalysts for Solar-to-Hydrogen Peroxide Energy Conversion with 0.2% Efficiency. *J Am Chem Soc* **138**, 10019-10025 (2016).
19. Kofuji Y*, et al.* Hydrogen Peroxide Production on a Carbon Nitride–Boron Nitride-Reduced Graphene Oxide Hybrid Photocatalyst under Visible Light. *ChemCatChem* **10**, 2070-2077 (2018).
20. Ye YX*, et al.* Highly efficient photosynthesis of hydrogen peroxide in ambient conditions. *Proc Natl Acad Sci U S A* **118**, (2021).
21. Cao J*, et al.* Phosphorus-doped porous carbon nitride for efficient sole production of hydrogen peroxide via photocatalytic water splitting with a two-channel pathway. *Journal of Materials Chemistry A* **8**, 3701-3707 (2020).
22. Cai J*, et al.* Crafting Mussel-Inspired Metal Nanoparticle-Decorated Ultrathin Graphitic Carbon Nitride for the Degradation of Chemical Pollutants and Production of Chemical Resources. *Adv Mater* **31**, e1806314 (2019).
23. Li S*, et al.* Effective photocatalytic H_2O_2 production under visible light irradiation at g-C3N4 modulated by carbon vacancies. *Applied Catalysis B: Environmental* **190**, 26-35 (2016).
24. Zhao S, Zhao X, Zhang H, Li J, Zhu Y. Covalent combination of polyoxometalate and graphitic carbon nitride for light-driven hydrogen peroxide production. *Nano Energy* **35**, 405-414 (2017).
25. Ma P*, et al.* Band alignment of homojunction by anchoring CN quantum dots on g-C_3N_4 (0D/2D) enhance photocatalytic hydrogen peroxide evolution. *Applied Catalysis B: Environmental* **300**, (2022).
26. Liu B*, et al.* Boosting O_2 Reduction and H_2O Dehydrogenation Kinetics: Surface N-Hydroxymethylation of g-C_3N_4 Photocatalysts for the Efficient Production of H2O2. **32**, 2111125 (2022).
27. Chu C*, et al.* Spatially separating redox centers on 2D carbon nitride with cobalt single atom for photocatalytic H_2O_2 production. *Proc Natl Acad Sci U S A* **117**, 6376-6382

(2020).

28. Zhao S, Zhao X. Polyoxometalates-derived metal oxides incorporated into graphitic carbon nitride framework for photocatalytic hydrogen peroxide production under visible light. *Journal of Catalysis* **366**, 98-106 (2018).
29. Chen L, Chen C, Yang Z, Li S, Chu C, Chen B. Simultaneously Tuning Band Structure and Oxygen Reduction Pathway toward High-Efficient Photocatalytic Hydrogen Peroxide Production Using Cyano-Rich Graphitic Carbon Nitride. *Advanced Functional Materials* **31**, (2021).
30. Zhu Z, Pan H, Murugananthan M, Gong J, Zhang Y. Visible light-driven photocatalytically active g-C_3N_4 material for enhanced generation of H2O2. *Applied Catalysis B: Environmental* **232**, 19-25 (2018).
31. Shiraishi Y*, et al.* Resorcinol-formaldehyde resins as metal-free semiconductor photocatalysts for solar-to-hydrogen peroxide energy conversion. *Nat Mater* **18**, 985-993 (2019).
32. Ma J*, et al.* Extended Conjugation Tuning Carbon Nitride for Non-sacrificial H_2O_2 Photosynthesis and Hypoxic Tumor Therapy. *Angew Chem Int Ed Engl* **61**, e202210856 (2022).
33. Chen L*, et al.* Acetylene and Diacetylene Functionalized Covalent Triazine Frameworks as Metal-Free Photocatalysts for Hydrogen Peroxide Production: A New Two-Electron Water Oxidation Pathway. *Adv Mater* **32**, e1904433 (2020).
34. Liu L, Gao MY, Yang H, Wang X, Li X, Cooper AI. Linear Conjugated Polymers for Solar-Driven Hydrogen Peroxide Production: The Importance of Catalyst Stability. *J Am Chem Soc* **143**, 19287-19293 (2021).
35. Cheng H, Lv H, Cheng J, Wang L, Wu X, Xu H. Rational Design of Covalent Heptazine Frameworks with Spatially Separated Redox Centers for High-Efficiency Photocatalytic Hydrogen Peroxide Production. *Adv Mater* **34**, e2107480 (2022).
36. Zhao C*, et al.* Molecular Level Modulation of Anthraquinone-containing Resorcinol-formaldehyde Resin Photocatalysts for H_2O_2 Production with Exceeding 1.2% Efficiency. *Angew Chem Int Ed Engl*, (2022).
37. Shiraishi Y, Matsumoto M, Ichikawa S, Tanaka S, Hirai T. Polythiophene-Doped Resorcinol-Formaldehyde Resin Photocatalysts for Solar-to-Hydrogen Peroxide Energy Conversion. *J Am Chem Soc* **143**, 12590-12599 (2021).
